# The burden of mental disorders in the Eastern Mediterranean region, 1990–2015: findings from the global burden of disease 2015 study

**DOI:** 10.1007/s00038-017-1006-1

**Published:** 2017-08-03

**Authors:** GBD 2015 Eastern Mediterranean Region Mental Health Collaborators, Raghid Charara, Charbel El Bcheraoui, Ibrahim Khalil, Maziar Moradi-Lakeh, Ashkan Afshin, Nicholas J. Kassebaum, Michael Collison, Kristopher J. Krohn, Adrienne Chew, Farah Daoud, Fiona J. Charlson, Danny Colombara, Louisa Degenhardt, Rebecca Ehrenkranz, Holly E. Erskine, Alize J. Ferrari, Michael Kutz, Janni Leung, Damian Santomauro, Haidong Wang, Harvey A. Whiteford, Amanuel Alemu Abajobir, Foad Abd-Allah, Haftom Niguse Abraha, Laith J. Abu-Raddad, Aliasghar Ahmad Kiadaliri, Alireza Ahmadi, Kedir Yimam Ahmed, Muktar Beshir Ahmed, Faris Hasan Al Lami, Khurshid Alam, Deena Alasfoor, Reza Alizadeh-Navaei, Juma M Alkaabi, Fatma Al-Maskari, Rajaa Al-Raddadi, Khalid A. Altirkawi, Nahla Anber, Hossein Ansari, Hamid Asayesh, Rana Jawad Asghar, Tesfay Mehari Atey, Tadesse Awoke Ayele, Till Bärnighausen, Umar Bacha, Aleksandra Barac, Suzanne L. Barker-Collo, Bernhard T. Baune, Shahrzad Bazargan-Hejazi, Neeraj Bedi, Isabela M. Bensenor, Adugnaw Berhane, Addisu Shunu Beyene, Zulfiqar A. Bhutta, Dube Jara Boneya, Rohan Borschmann, Nicholas J. K. Breitborde, Zahid A Butt, Ferrán Catalá-López, Liliana G. Ciobanu, Hadi Danawi, Amare Deribew, Samath D. Dharmaratne, Kerrie E. Doyle, Aman Yesuf Endries, Emerito Jose Aquino Faraon, André Faro, Maryam S. Farvid, Wubalem Fekadu, Seyed-Mohammad Fereshtehnejad, Florian Fischer, Tsegaye Tewelde Gebrehiwot, Ababi Zergaw Giref, Melkamu Dedefo Gishu, Alessandra Carvalho Goulart, Tesfa Dejenie Habtewold, Randah Ribhi Hamadeh, Mitiku Teshome Hambisa, Samer Hamidi, Josep Maria Haro, Mohammad Sadegh Hassanvand, Nobuyuki Horita, Mohamed Hsairi, Hsiang Huang, Abdullatif Husseini, Mihajlo B. Jakovljevic, Spencer Lewis James, Jost B. Jonas, Amir Kasaeian, Yousef Saleh Khader, Ejaz Ahmad Khan, Abdullah Tawfih Abdullah Khoja, Ardeshir Khosravi, Jagdish Khubchandani, Daniel Kim, Yun Jin Kim, Yoshihiro Kokubo, Ai Koyanagi, Barthelemy Kuate Defo, Heidi J. Larson, Asma Abdul Latif, Paul H. Lee, Cheru Tesema Leshargie, Ricky Leung, Loon-Tzian Lo, Raimundas Lunevicius, Hassan Magdy Abd El Razek, Mohammed Magdy Abd El Razek, Reza Majdzadeh, Azeem Majeed, Reza Malekzadeh, Jose Martinez-Raga, Habibolah Masoudi Farid, Mohsen Mazidi, John J. McGrath, Ziad A. Memish, Walter Mendoza, Melkamu Merid Mengesha, Mubarek Abera Mengistie, Haftay Berhane Mezgebe, Ted R. Miller, Philip B. Mitchell, Alireza Mohammadi, Shafiu Mohammed, Carla Makhlouf Obermeyer, Felix Akpojene Ogbo, Elizabeth Palomares Castillo, Christina Papachristou, Scott B. Patten, George C. Patton, Aslam Pervaiz, Michael Robert Phillips, Farshad Pourmalek, Mostafa Qorbani, Amir Radfar, Anwar Rafay, Vafa Rahimi-Movaghar, Rajesh Kumar Rai, David Laith Rawaf, Salman Rawaf, Amany H. Refaat, Satar Rezaei, Mohammad Sadegh Rezai, Gholamreza Roshandel, Mahdi Safdarian, Mahdi Safiabadi, Saeid Safiri, Rajesh Sagar, Mohammad Ali Sahraian, Payman Salamati, Abdallah M. Samy, Benn Sartorius, Mete I. Saylan, Soraya Seedat, Sadaf G. Sepanlou, Masood Ali Shaikh, Morteza Shamsizadeh, Diego Augusto Santos Silva, Jasvinder A. Singh, Badr H. A. Sobaih, Dan J. Stein, Rizwan Suliankatchi Abdulkader, Bryan L. Sykes, Rafael Tabarés-Seisdedos, Karen M. Tabb, Arash Tehrani-Banihashemi, Mohamad-Hani Temsah, Abdullah Sulieman Terkawi, Roman Topor-Madry, Kingsley Nnanna Ukwaja, Olalekan A. Uthman, Stein Emil Vollset, Tolassa Wakayo, Yuan-Pang Wang, Andrea Werdecker, Ronny Westerman, Abdulhalik Workicho, Mohsen Yaghoubi, Hassen Hamid Yimam, Naohiro Yonemoto, Mustafa Z. Younis, Chuanhua Yu, Maysaa El Sayed Zaki, Bassel Zein, Aisha O. Jumaan, Theo Vos, Simon I. Hay, Mohsen Naghavi, Christopher J. L. Murray, Ali H. Mokdad

**Affiliations:** Institute for Health Metrics and Evaluation, University of Washington, Seattle, WA USA

**Keywords:** Mental health, Eastern Mediterranean region, Burden of disease, Depressive disorders, Anxiety disorders

## Abstract

**Objectives:**

Mental disorders are among the leading causes of nonfatal burden of disease globally.

**Methods:**

We used the global burden of diseases, injuries, and risk factors study 2015 to examine the burden of mental disorders in the Eastern Mediterranean region (EMR). We defined mental disorders according to criteria proposed in the diagnostic and statistical manual of mental disorders IV and the 10th International Classification of Diseases.

**Results:**

Mental disorders contributed to 4.7% (95% uncertainty interval (UI) 3.7–5.6%) of total disability-adjusted life-years (DALYs), ranking as the ninth leading cause of disease burden. Depressive disorders and anxiety disorders were the third and ninth leading causes of nonfatal burden, respectively. Almost all countries in the EMR had higher age-standardized mental disorder DALYs rates compared to the global level, and in half of the EMR countries, observed mental disorder rates exceeded the expected values.

**Conclusions:**

The burden of mental disorders in the EMR is higher than global levels, particularly for women. To properly address this burden, EMR governments should implement nationwide quality epidemiological surveillance of mental disorders and provide adequate prevention and treatment services.

**Electronic supplementary material:**

The online version of this article (doi:10.1007/s00038-017-1006-1) contains supplementary material, which is available to authorized users.

## Introduction

Mental illness is a growing public health concern. Findings from Global Burden of Diseases, Injuries, and Risk Factors Study 2015 (GBD 2015) showed that mental disorders are among the highest ranking causes of nonfatal burden globally (GBD 2015 Disease and Injury Incidence and Prevalence Collaborators 2016). More specifically, depressive disorders and anxiety disorders were the third and ninth leading contributors to years lived with disability (YLDs)—a measure of nonfatal burden. The global prevalence and nonfatal burden of mental disorders were 905,733,400 cases and 124,193,900 YLDs, respectively. Five percent of global DALYs and 15.7% of global YLDs were due to mental disorders (Kassebaum et al. 2016). One DALY represents the loss of a healthy year of life and aggregates the YLDs with the years of life lost (YLLs) due to premature mortality.

The EMR is a World Health Organization (WHO)-defined group of countries comprising Afghanistan, the Arab Republic of Egypt (Egypt), Bahrain, Djibouti, Iraq, the Islamic Republic of Iran (Iran), Jordan, the Kingdom of Saudi Arabia (Saudi Arabia), Kuwait, Lebanon, Libya, Morocco, Oman, Pakistan, Palestine, Qatar, the Republic of Yemen (Yemen), Somalia, Sudan, the Syrian Arab Republic (Syria), Tunisia, and the United Arab Emirates (UAE). Its population was estimated to be 628 million in 2014 (World Health Organization Regional Office for the Eastern Mediterranean 2014). The EMR is a very heterogeneous region where member states vary significantly in terms of their gross domestic product, sociodemographic profiles, health indicators, and health system capacities and coverage (Mandil et al. 2013).

Over the past two decades, the EMR has undergone significant improvements in health status, including increased life expectancy and reductions in child mortality (Memish 2014; Mokdad et al. 2014, 2016a; Moradi-Lakeh et al. 2016). As people in the region are living longer, the burden of chronic diseases, including mental disorders, is expected to rise. Increasing mental disorder burden with population aging, especially in developing countries, has been described in the literature (Sathyanarayana Rao and Shaji 2007; World Health Organization 2016). The demographic and epidemiological changes in the EMR have had a major impact on the organization and delivery of mental health services. The population in EMR is very young, where the median age is about 23 years, around 60% of the population is between 15 and 59 years of age, and one third is below 15 years of age (World Health Organization 2013a).

Moreover, 85% of the EMR population is or has been (in the past quarter century) in a complex emergency situation resulting in a high prevalence of depression, anxiety, and post-traumatic stress disorder (Ghosh et al. 2004). Since 2010, the region has witnessed economic and political unrest (Mokdad et al. 2016b). The latter were seen in Egypt, Libya, Tunisia, and Yemen. Currently, Syria is in a state of civil war. Afghanistan, Bahrain, Iraq, Palestine, Lebanon and Somalia frequently experience disturbances as well. Conflict predisposes a population to the development of mental disorders (Murthy and Lakshminarayana 2006). Stressors of war include loss (human or material) and grief, safety concerns, disruption of the fabric of society, loss of identity, and potential discrimination with displacement (Hassan et al. 2016).

No previous studies have assessed the burden of mental disorders in the EMR collectively. Using data from GBD 2015, we aim to explore the burden of mental disorders in the EMR by country, age group, sex, type of mental disorder, and income group from 1990 to 2015. We have previously published a study on the burden of mental disorders in the EMR between 1990 and 2013 using findings from GBD 2013. We now update the burden estimates using a wide range of updated and standardized analytical procedures.

## Methods

### Case definition

In this study, we present GBD 2015 results for mental disorders, excluding substance use disorders. The GBD 2015 mental disorders grouping consisted of anxiety disorders, autistic spectrum disorders (autism and Asperger’s syndrome), conduct disorder, eating disorders (anorexia nervosa and bulimia nervosa), schizophrenia, attention-deficit/hyperactivity disorder (ADHD), bipolar disorder, depressive disorders (major depressive disorder and dysthymia), and idiopathic developmental intellectual disability (a residual category capturing intellectual disability not attributed to any of the other causes in the study). We defined mental disorders according to criteria proposed in the Diagnostic and Statistical Manual of Mental Disorders IV (DSM-IV) and the International Classification of Diseases 10 (ICD-10) (GBD 2015 Disease and Injury Incidence and Prevalence Collaborators 2016). The DSM-IV and ICD-10 definitions of the mental disorders described in this study are published in detail in the GBD 2015 nonfatal burden capstone study (GBD 2015 Disease and Injury Incidence and Prevalence Collaborators 2016).

### Calculation of burden (YLDs)

The estimation of YLDs for a given disorder is a product of epidemiological data that accommodates the number of people affected as well as the severity and disability associated with their symptoms. That is, YLDs are calculated by multiplying the prevalence of a disorder by its severity and comorbidity-adjusted disability weight. YLDs for previous GBD iterations were re-estimated using the same methods to allow meaningful comparisons of changes over time.

#### Epidemiologic inputs

Prevalence, incidence, remission or duration, and excess mortality data for mental disorders were captured through a systematic review of the literature. In GBD 2010, a literature search was conducted in three stages involving electronic searches of the peer‐reviewed literature (via Medline, Embase and PubMed), gray literature, and expert consultation. The agreed approach for mental disorders was to conduct electronic database searches on a rolling basis. All three stages of the GBD 2010 literature review were repeated for GBD 2013 to capture additional data published up to 2013. For GBD 2015, only stages two and three of the literature review were conducted, with another electronic database search due for mental disorders in the next iteration of GBD studies. The inclusion criteria for epidemiologic studies stipulated that: (1) the publication year must be from 1980 onward; (2) “caseness” must be based on clinical threshold as established by the DSM-IV or ICD-10; (3) sufficient information must be provided on study method and sample characteristics to assess the quality of the study; and (4) study samples must be representative of the general population (i.e., inpatient or pharmacological treatment samples, case studies, veterans or refugee samples were excluded). No limitation was set on the language of publication. Methods used for this systematic review have been reported in greater detail elsewhere (GBD 2015 Disease and Injury Incidence and Prevalence Collaborators 2016). Data from 108 epidemiologic studies were used to estimate the burden of mental disorders in the EMR in GBD 2015; a full list of the studies is available in Appendix 1 in supplementary.

#### Disease modeling

For each disorder, epidemiological estimates from the literature review were pooled using DisMod-MR 2.1, a Bayesian meta-regression tool. The tool used in GBD 2010 and GBD 2013, DisMod-MR, is based on a generalized negative binomial model that: (1) uses an incidence–prevalence–mortality mathematical model to enforce internal consistency between estimates from different epidemiological parameters; (2) estimates data for countries and world regions with few or no available input data based on random effects for country, regions, and their corresponding super-region groupings; (3) deals with variability in the data due to measurement bias, or alternatively, ecological factors through the use of study- and country-level covariates; and (4) propagates uncertainty around the raw epidemiological data through to the final estimates. For GBD 2015, the computational engine of DisMod-MR 2.1 remained unchanged, but we substantially rewrote the code that organizes the flow of data and settings at each level of the analytical cascade. Greater detail on DisMod-MR 2.1 is available elsewhere (GBD 2015 Disease and Injury Incidence and Prevalence Collaborators 2016).

#### Disability weights

Disability weights are the general public’s assessment of the severity of health loss associated to the cause. Disability weights were derived using the GBD 2010 method of pairwise comparison questions in population surveys (of those aged 18 and over) conducted in Bangladesh, Indonesia, Peru, Tanzania, Hungary, Italy, Sweden, Netherlands, and the United States and an open access internet survey. Respondents considered two hypothetical individuals with different health states and were asked to indicate which person they perceived as healthier. In GBD 2013, methodological advances were introduced to disability weighting, including new data capturing many newly published or unpublished data sources for the disorders included in GBD. A disability weight ranging between 0 (equivalent to perfect health) and 1 (equivalent to death) was generated for 235 health states which together reflected all causes of nonfatal burden in GBD 2015 (Salomon et al. 2015; GBD 2015 Disease and Injury Incidence and Prevalence Collaborators 2016).

#### Severity distributions

Sequelae were further defined in terms of severity following the same approach for estimating the distribution of severity as in GBD 2013. Details on the severity distributions for mental disorders are available elsewhere (GBD 2015 Disease and Injury Incidence and Prevalence Collaborators 2016).

#### Comorbidity adjustment

GBD 2015 YLD estimates were adjusted for the effect of comorbidity between diseases. Details on the process are available elsewhere (GBD 2015 Disease and Injury Incidence and Prevalence Collaborators 2016).

### Calculation of burden (DALYs)

We calculated DALYs as the sum of YLDs and YLLs. YLLs were calculated by multiplying the number of deaths due to the given disorder at a particular age by the standard life expectancy at that age. However, death records used in GBD 2015 followed ICD-10 rules for categorical attribution of cause of death to a single underlying cause and, therefore, did not document any deaths due to mental disorders, except for schizophrenia and eating disorders.

### Socio-demographic index

In GBD 2015, we constructed a summary metric referred to as the Socio-demographic Index (SDI) based on measures of income per capita, average years of schooling among people aged 15 years and older, and total fertility rate (Kassebaum et al. 2016). SDI values were scaled to a range of 0–1, with 0 equaling the lowest income, lowest schooling, and highest fertility rate observed from 1980 to 2015, and 1 equaling the highest income, highest schooling, and lowest fertility rate observed during that time. The final SDI score was computed as the geometric mean of each of the components. We compared observed patterns of mental disorder YLDs with those expected on the basis of SDI, allowing us to explore where health gains exceeded—or lagged behind-corresponding changes in development.

### Classification of EMR countries

In an attempt to properly track the health status in the EMR countries, we divided the region into three categories according to the gross national income (GNI) per capita. The first category represented the low-income countries (LICs) with an average GNI per capita of $523 (GBD 2015 Disease and Injury Incidence and Prevalence Collaborators 2016; GBD 2015 Maternal Mortality Collaborators 2016; GBD 2015 Mortality and Causes of Death Collaborators 2016; GBD 2015 Risk Factors Collaborators 2016; Kassebaum et al. 2016; Wang et al. 2016). On the opposite end of the spectrum were some oil-rich, high-income countries (HICs) with an average GNI per capita of $39,688. The nations that lied in between were the middle-income countries (MICs) with an average GNI per capita of $3,251. The three groups were LICs: Afghanistan, Djibouti, Yemen, and Somalia; MICs: Egypt, Iraq, Iran, Jordan, Lebanon, Libya, Morocco, Pakistan, Palestine, Sudan, Syria, and Tunisia; and HICs: Bahrain, Saudi Arabia, Kuwait, Oman, Qatar, and the UAE.

## Results

In 2015, the EMR generated a total of 229.2 million DALYs (95% uncertainty interval (UI) 194.8–267.6) of which 10.7 million DALYs (7.1–15.0) were due to mental disorders. In other words, mental disorders contributed to 4.7% (3.7–5.6%) of total DALYs in the EMR in 2015, ranking as the ninth leading cause of disease burden.

In 2015, the EMR generated a total of 67.1 million YLDs (46.9–91.2) of which 10.7 million YLDs (7.1–14.9 million) were due to mental disorders. In other words, mental disorders contributed to 15.9% (15.1–16.4%) of total YLDs in the EMR in 2015, ranking second to musculoskeletal disorders which contributed to 16.1% (14.2–18.2%) of total YLDs. At a more detailed level, depressive disorders and anxiety disorders were the third and ninth leading causes of YLDs, respectively.

**Fig. 1:**
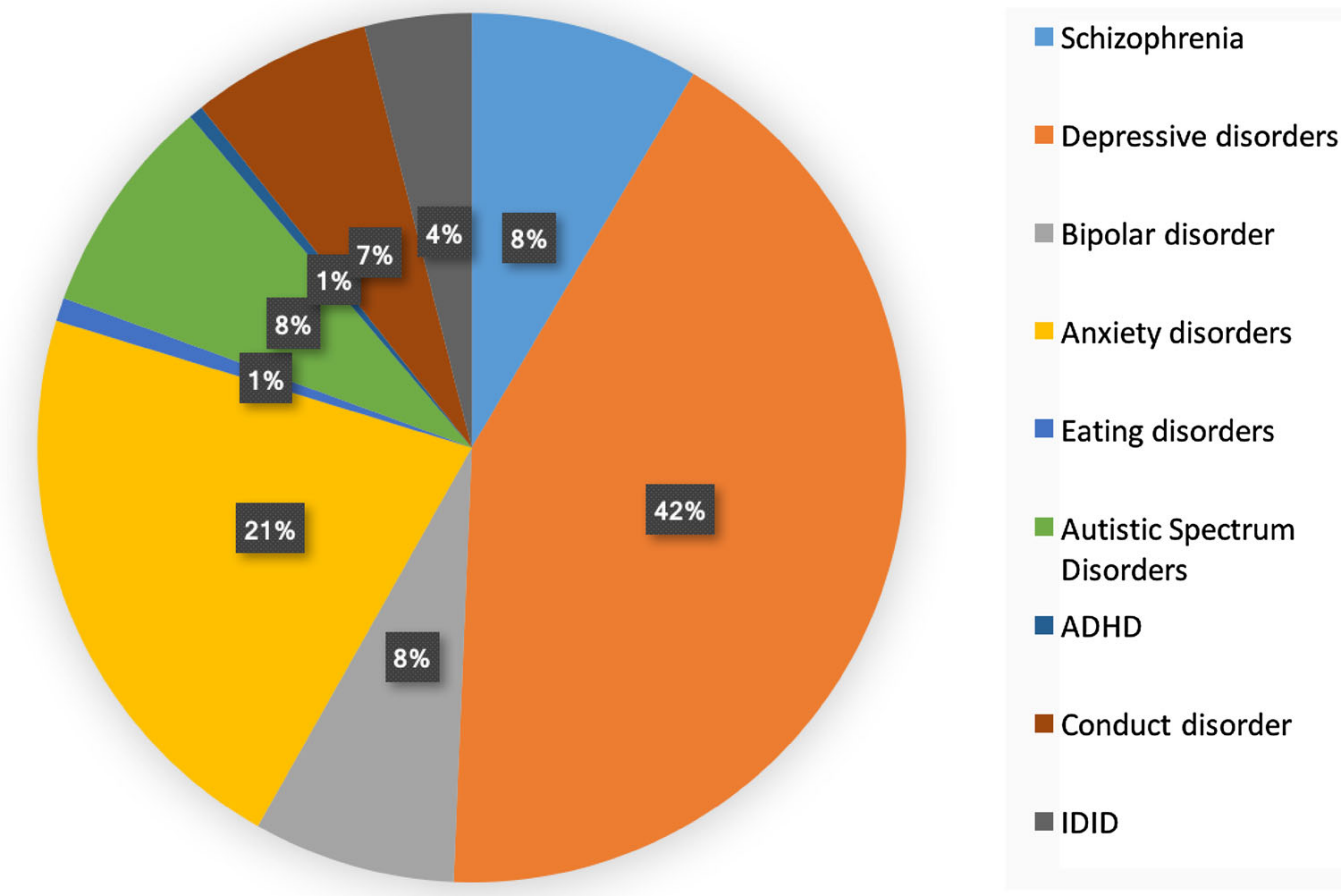
Distribution of disability-adjusted life-years (DALYs) due to mental disorders in the Eastern Mediterranean Region, 2015. (Global Burden of Disease Study 2015, Eastern Mediterranean Region, 2015)

Figure 1 shows the composition of mental disorder DALYs by type of disorder for both sexes combined in the EMR in 2015. Depressive disorders (42.1%), followed by anxiety disorders (21.5%), were the greatest contributors to mental disorder DALY numbers.

**Fig. 2:**
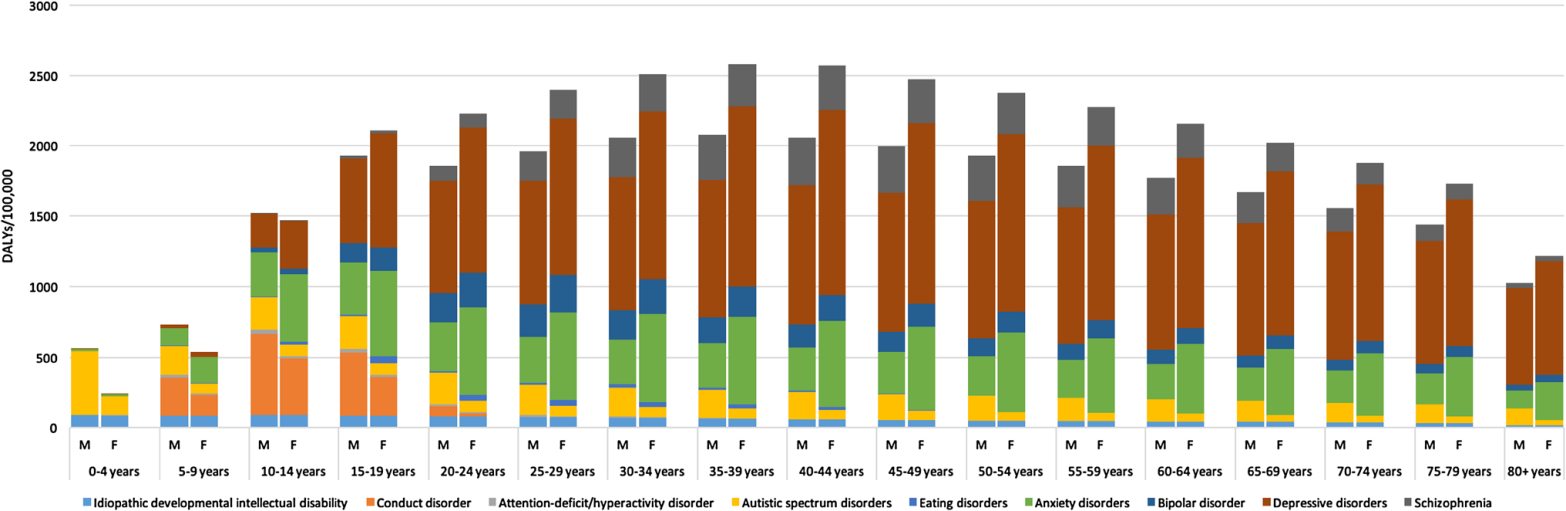
Age-standardized rate of disability-adjusted life-years (DALYs) per 100,000 population due to mental disorders in the Eastern Mediterranean Region by age, sex, and disorder, 2015. (Global Burden of Disease Study 2015, Eastern Mediterranean Region 2015)

Figure 2 shows the composition of mental disorder DALY rates by age, sex, and disorder type in the EMR in 2015. DALY rates were higher for females across all age groups, except for those under age 15. The highest rate of DALYs occurred in the 25–49 age group, with a peak in the 35–39 age group for both sexes combined, females, and males. The age pattern in males and females was different. In females, DALY rates increased progressively from birth up to age 35–39 (3131 DALYs/100,000) and then decreased progressively with age. In males, DALY rates peaked at age 15–19 (1929 DALYs/100,000) and decreased at age 20–24 (1860 DALYs/100,000), only to rise again and peak at age 35–39 (2075 DALYs/100,000). The burden associated with depressive disorders and anxiety disorders rose abruptly in adolescence for both sexes. Depressive disorder burden peaked between 40 and 44 years, whereas anxiety disorder burden peaked between 15 and 19 years. For schizophrenia, the burden peaked between 40 and 49 years while bipolar disorder peaked between 25 and 29 years. Conduct disorder and ADHD peaked between 10 and 14 years. Supplementary e-Table 1 details the values of the DALY rates depicted in the figure.

**Fig. 3:**
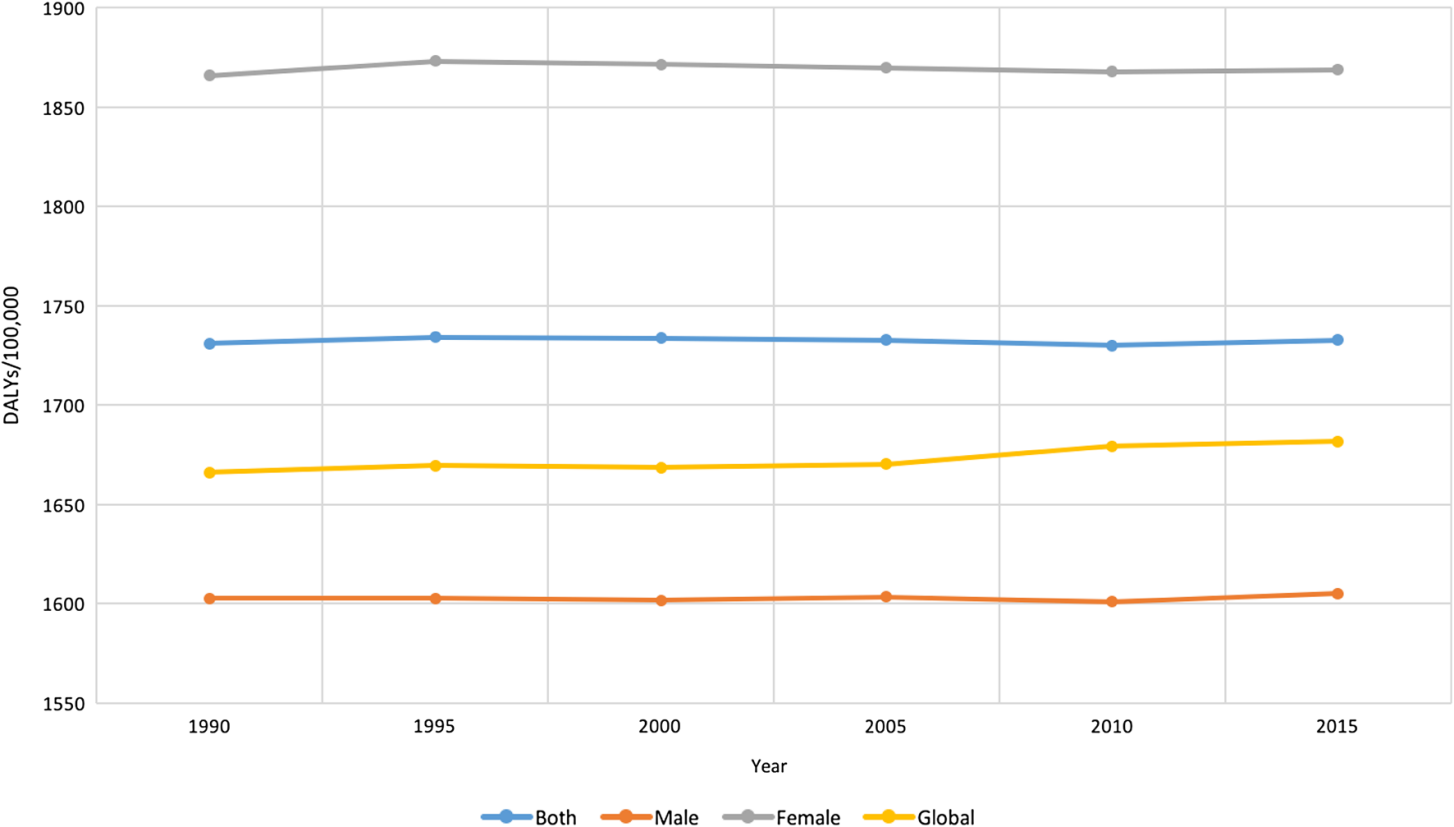
Age-standardized rate of disability-adjusted life-years (DALYs) per 100,000 population due to mental disorders in the Eastern Mediterranean Region and globally, 1990–2015. (Global Burden of Disease Study 2015, Global, Eastern Mediterranean Region 1990–2015)

Figure 3 shows the age-standardized rate of mental disorder DALYs over time for males, females, and both combined in the EMR and globally. The rate of DALYs remained almost constant from 1990 to 2015 in the EMR for both males and females and globally. EMR DALY rates in females were consistently higher than their male counterparts and in the EMR compared to the global rates from 1990 to 2015. The rate of DALYs in males was lower than the global level. Supplementary e-Table 2 details the values of DALY rates depicted in the figure. None of these differences were statistically significant. Table 1 shows the rankings (in age-standardized DALY rates) of each mental disorder in the EMR in 2015 compared to 1990. All mental disorders ranked higher in 2015.

**Table 1:** Ranking of mental disorders among all level 3 Global Burden of Disease (GBD) causes for age-standardized rates of disability-adjusted life-years, 1990–2015

	1990 rank	2015 rank
Schizophrenia	69	56
Idiopathic developmental intellectual disability	105	92
Bipolar disorder	77	65
Conduct disorder	86	74
Autistic spectrum disorders	73	62
Eating disorders	147	140
Anxiety disorders	33	27
Depressive disorders	16	14
Attention-deficit/hyperactivity disorder	150	149

Global Burden of Disease Study 2015, Eastern Mediterranean Region 2015

**Fig. 4:**
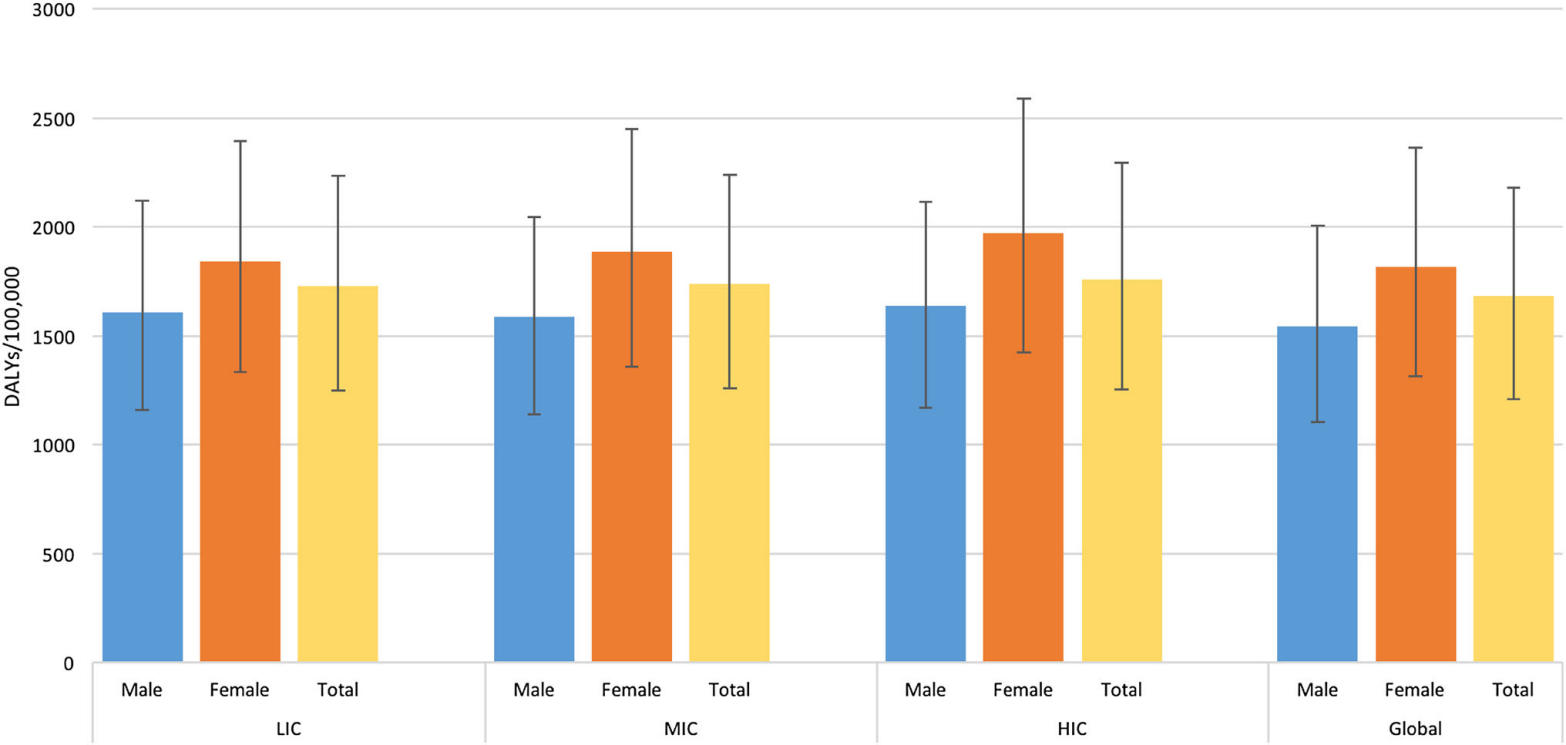
Age-standardized rate of disability-adjusted life-years (DALYs) due to mental disorders in the Eastern Mediterranean Region and Globally, by income group and sex, 2015. (Global Burden of Disease Study 2015, Global, Eastern Mediterranean Region, 2015)

Figure 4 shows the rate of mental disorder DALYs in the EMR in 2015 by income grouping and sex. The DALY rates were similar across income groups, ranging from 1725.9 DALYs/100,000 in LIC to 1758.2 DALYs in HIC. Across these groups, females had higher mental disorder DALY rates compared to males. All EMR income groups had higher rates of DALYs compared to global levels. Supplementary e-Table 3 details the values of DALY rates depicted in the figure. None of these differences were statistically significant.

**Fig. 5:**
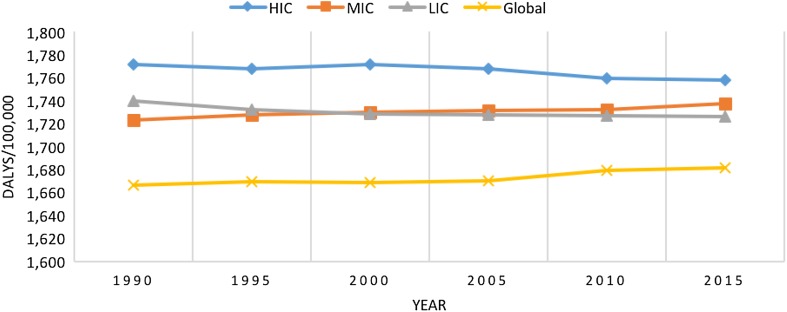
Age-standardized rate of disability-adjusted life-years (DALYs) due to mental disorders in the Eastern Mediterranean Region and globally by income group, 1990–2015. (Global Burden of Disease Study 2015, Global, Eastern Mediterranean Region, 1990–2015)

Figure 5 shows the age-standardized rate of mental disorder DALYs in the EMR and globally over time, by income grouping. The DALY rates remained almost constant in the EMR income groups and globally. Differences between income groups and over time were not statistically significant.

e-Figure 1 shows the age-standardized rate of mental disorder DALYs in the EMR, by country and sex. The countries are ranked in increasing order of age-standardized mental disorder DALY rates for both sexes combined. All countries in the EMR, except Egypt, had higher age-standardized mental disorder DALY rates compared to the global value.

e-Figure 2 shows the age-standardized observed mental disorder DALY rates by EMR country compared with the expected values on the basis of each country’s SDI. For the EMR, SDI in 2015 ranged from 0.151 for Somalia to 0.875 for the UAE. The area between the observed and expected curves represents the discrepancy between both values (∆ = observed−expected). The countries are ranked in the order of decreasing ∆. In half of the EMR countries, observed mental disorder DALYs exceeded the expected values.

## Discussion

Our study reveals a number of findings that are relevant to setting a mental health agenda for the EMR. The burden associated with mental disorders has not seen a significant drop in the EMR (and globally) over the last quarter century, and females continue to suffer a bigger burden of mental illness. The mental disorder burden in the politically and economically stable high-income countries is comparable to that in low- and middle-income countries (LMICs) of the region, which include some countries in complex emergency situations. Almost all countries in the EMR have a bigger burden of mental disorders compared to the global rate.

### Epidemiological transition

While the rate of mental disorder DALYs has not significantly changed over time, the ranking of these disorders shows a different picture. Compared to 1990, there was an increase in the amount of burden mental disorders contribute to the total burden. This epidemiological transition is consistent with that seen globally, especially at higher SDIs (GBD 2015 Disease and Injury Incidence and Prevalence Collaborators 2016). The increasing mental health burden is mainly attributable to population growth and aging rather than an increase in prevalence rates. This is an important finding to be considered when setting public health agendas for the region as more people will be experiencing mental disorders and for longer durations.

### Women’s mental health

Females have a higher burden of mental disorders in the EMR. Rates of mental disorders, however, only partially explain gender disparities in mental health. Risk factors, timing of illness onset, diagnosis, treatment, and adjustment to a chronic illness are to be accounted for (World Health Organization 2006). Women in this part of the world are particularly vulnerable to developing mental illness, namely depressive disorders. With globalization and urbanization of most EMR societies, women may be exposed to numerous stressors. This distress may have repercussions on the entire household and children in particular, as adjustments to new social structures become necessary (Eloul et al. 2009). According to the World Bank, there has been a 47% increase in women’s participation in the labor market of the Middle East and North Africa region from 1960 to 2000 (World Bank 2003). Gender roles in the region may partially contribute to a higher burden of mental illness among women. Socioeconomic disadvantage, low income and income inequality, low or subordinate social status and rank, and lack of autonomy are some examples of this polarity (World Health Organization 2006). The impact of patriarchy and women’s lack of empowerment on mental health is thoroughly evaluated in the literature (Niaz and Hassan 2006). Moreover, cultural factors, high birthrates, and young ages at first conception may contribute to higher rates of postpartum depression (Eloul et al. 2009). Perinatal mental disorders are particularly concerning for their effects on the development of infants and children (World Health Organization 2010). In addition, the region witnesses one of the highest rates (37.0%) of intimate partner violence in the world (compared to 25.4% in Europe and 29.8% in the Americas) (World Health Organization 2013b). Women are also more likely to be diagnosed with depression compared to men with similar scores on standardized measures of depression (Callahan et al. 1997; Stoppe et al. 1999).

### The burden by disorder type

Depression is the highest contributor to the burden followed by anxiety disorders. This is because of the higher prevalence of these disorders as well as the disability associated with them. It is worth mentioning that some highly disabling conditions, such as acute schizophrenia, do not rank high in YLDs owing to their low prevalence (Ferrari et al. 2014). This latter condition, however, had the highest disability weight (0.778) among mental disorders in GBD 2015 (GBD 2015 Disease and Injury Incidence and Prevalence Collaborators 2016). Under the age of 15, males contribute more greatly to the burden of mental disorders, primarily due to the higher burden of autism in males under age five and conduct disorder in males ages 5–15. Indeed, epidemiologic studies from the region indicate a high male to female ratio of autistic spectrum disorders (Elsabbagh et al. 2012).

### Observed vs expected rates

According to GBD 2015 findings, sizeable discrepancies occurred for observed and expected YLDs based on SDI throughout North Africa and the Middle East, probably reflecting the uneven achievements in development found in this region (GBD 2015 Disease and Injury Incidence and Prevalence Collaborators 2016). At a regional level, observed depression YLD rates exceeded expected rates based on SDI. This implies that the EMR’s income per capita, educational levels and fertility rates were not commensurate with the high burden of depression seen in the region. High-income countries in the EMR had the highest SDIs in 2015 but had observed depression YLD rates that exceeded expected rates based on SDI. This is not consistent with the global trend where the proportion of life expectancy spent with disability declined slightly with increasing SDI (Kassebaum et al. 2016).

### Capacity of EMR versus burden

This high burden of mental disorders is particularly challenging to the EMR, where 16 out of the 22 countries in the region belong to the LMIC group. WHO’s 2014 Mental Health Atlas (World Health Organization 2014) described the preparedness of the EMR to deal with mental health at a system level. The mental health workforce per 100,000 population in the EMR was 7.3 compared to a value of 9 and 43.5 globally and in the WHO European region. A study published in 2005 showed that Lebanon had the highest provision of psychiatric services with one psychiatrist per 45,000 population (Al-Krenawi 2005). A more recent paper in 2012 reported the highest proportions of psychiatrists in Bahrain (5 per 100,000), Qatar (3.4 per 100,000) and Kuwait (3.1 per 100,000) (Okasha et al. 2012). Countries like Iraq, Libya, Morocco, Sudan, Syria, and Yemen had fewer than 0.5 psychiatrists per 100,000 population. It is important to note, however, that most of these studies had design weaknesses, meaning all outcomes must be interpreted with caution. The region’s WHO-Assessment Instrument for Mental Health Systems (World Health Organization Regional Office for the Eastern Mediterranean 2010) showed that the median-treated prevalence of mental disorders was 0.31%, thereby suggesting a big gap in treatment. The unmet needs of children and adolescents were greater than those of adults. This is alarming in the region where a big proportion of the population is under 19 years of age (World Health Organization 2010). Compared to the $3–$4 USD per capita spending on mental health in the US, the region spends an average of $0.15 USD per capita with only 2% of the governments’ health budgets allocated to mental health (which compares to the 5–10% required to match contemporary comprehensive healthcare systems) (Gater and Saeed 2015). This level of spending is observed across low-, middle- and high-income countries of the EMR. Not only are the resources scarce but also inefficiently used and inequitably distributed. Stigma further limits the use of available resources. Moreover, there is little integration of mental health in primary health care in much of the region (World Health Organization Regional Office for the Eastern Mediterranean 2010).

Some governments within the EMR have already taken action to address the problematic increase in mental disorder burden. Qatar has launched its 2013–2018 Mental Health Strategy which aims to increase availability and utilization of mental health services using comprehensive standards and guidelines (Supreme Council of Health, State of Qatar and Hamad Medical Corporation, Primary Health Care 2013). Kuwait is taking action to integrate mental health in primary health care in light of the stigma associated with mental illness and its impact on help seeking, especially in the EMR (Almazeedi and Alsuwaidan 2014). A study found that pharmacologic and/or psychosocial treatment packages can be offered at low prices in LMICs, including Morocco and Iran. It is important to point out that the development of psychosocial interventions needs to be tailored to the culture in the EMR. Most interventions used today in the region are simply exported from research in more developed nations instead of being adopted in a culturally sensitive fashion (Chisholm et al. 2007).

### Limitations

Our study has a number of limitations. First, our findings were based upon best available secondary data and models that cannot be verified across geographies or time within the same region due to historical circumstances and constraints of local resources. Most countries in the EMR lack quality epidemiological data to describe the national prevalence and burden of mental disorders and to provide quality representative data input for the GBD estimations. Raw prevalence data was available for 10 of the 22 EMR countries: Egypt, Iran, Iraq, Jordan, Lebanon, Pakistan, Palestine, Sudan, the UAE, and Yemen. A list of all data points used in this study are available via the Global Health Data Exchange (http://ghdx.healthdata.org). When data were of poor quality or unavailable, we relied on modeling techniques to generate the estimates using other available variables and the information for neighboring countries or countries with a similar health profile in the region. While this allowed us to include all EMR countries in our burden of disease analysis and generate collective measures for such a heterogeneous group of countries, it is important for countries in the region to facilitate the collection of high quality epidemiological data for mental disorders. Note, however, that the limitation of data availability is partly captured by the estimates of uncertainty presented in our results. Second, disability weights in GBD studies intentionally capture health loss while not attempting to capture welfare loss and hence do not reflect the economic and familial effects of mental disorders. In addition, given the subjective nature of the symptoms of mental illness, many individuals in cultures who express mental disorders differently from the ICD-10 diagnostic criteria were not captured by GBD. Third, deaths that were causally linked to mental disorders were largely captured under other causes. This is because an outcome could only be listed once in the GBD cause list. Vital registrations rarely list a mental disorder as a cause of death. For instance, major depressive disorder-related deaths from suicide or ischemic heart disease were captured under intentional injuries and cardiovascular disease, respectively. Fourth, DSM-IV and ICD-10 diagnostic criteria, mainly established in developed nations, may not be sensitive to all cross-cultural presentations of mental disorders. Many patients in the EMR attribute their psychiatric symptoms to physical causes, probably secondary to stigma. This would, therefore, bias the mental disorder burden estimates for the region. It is important to note here that none of the EMR countries were included in the group where surveys were done to estimate disability weights.

## Electronic supplementary material

Below is the link to the electronic supplementary material.

Supplementary material 1 (PDF 79 kb)

Supplementary material 2 (XLSX 29 kb)

Supplementary material 3 (DOCX 1189 kb)
