# Supplementary material for: The burden of mental disorders in the Eastern Mediterranean region, 1990–2015: findings from the global burden of disease 2015 study
Source: Int J Public Health. 2017 Aug 3;63(Suppl 1):25–37. doi: 10.1007/s00038-017-1006-1 (PMC5973970; doi:10.1007/s00038-017-1006-1)

Electronic Supplementary Material

**Article title:**

The Burden of Mental Disorders in the Eastern Mediterranean Region, 1990-2015: Findings from the Global Burden of Disease 2015 Study

**Journal:**

International Journal of Public Health

**Authors:**

GBD 2015 Eastern Mediterranean Region Mental Health Collaborators

**Corresponding author:**

Ali H. Mokdad

Institute for Health Metrics and Evaluation, University of Washington, Seattle, WA, United States

Email: [mokdaa@uw.edu](mailto:mokdaa@uw.edu)

e-Figure 1 – Age-standardized rates of disability-adjusted life-years (DALYs) per 100,000 population for mental disorders in the Eastern Mediterranean Region and Globally, by sex, in 2015. (Global Burden of Disease Study 2015, Global, Eastern Mediterranean Countries, 2015).


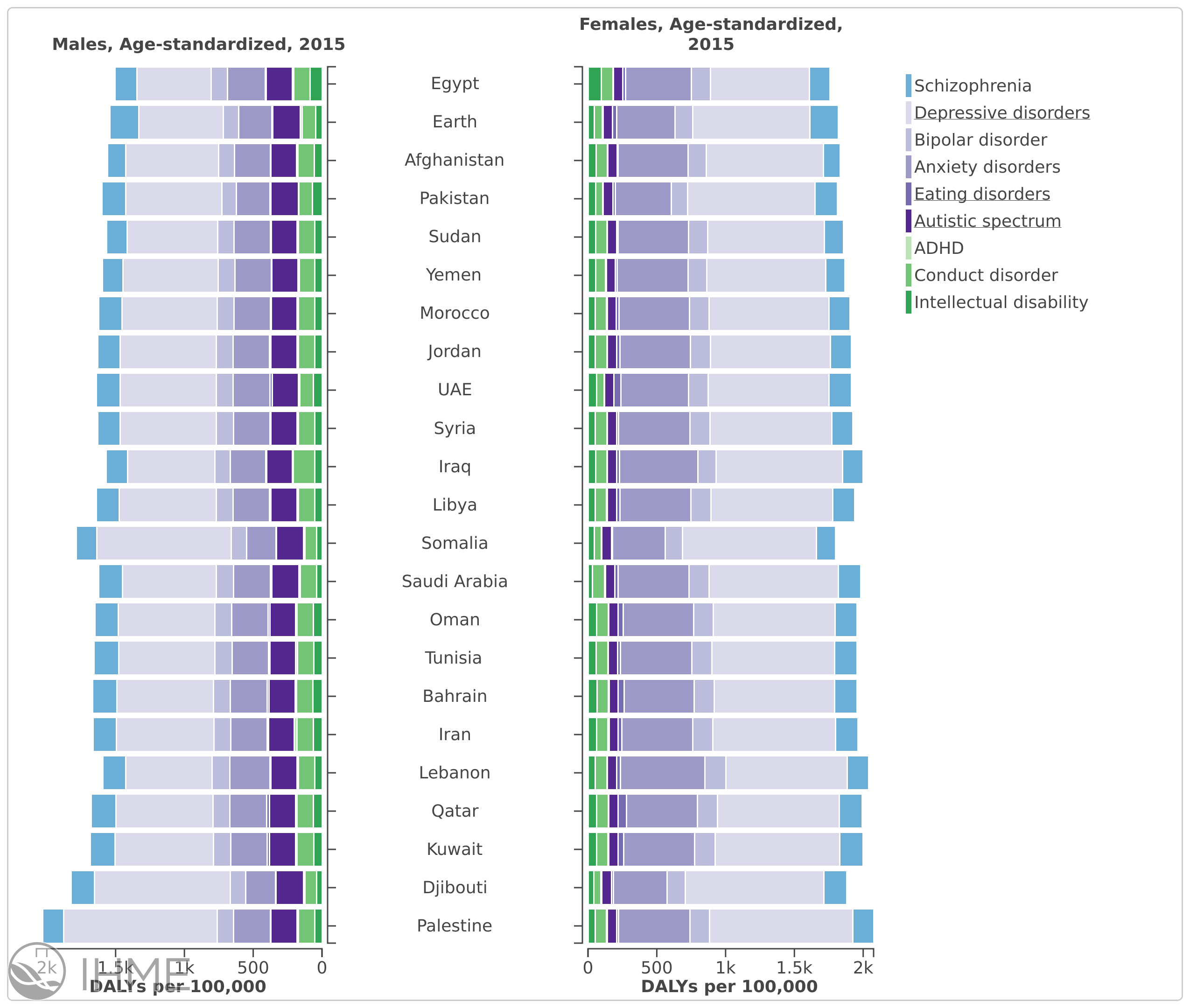


e-Figure 2 – Observed and expected age-standardized rates of disability-adjusted life-years(DALYs) per 100,000 population for mental disorders in the Eastern Mediterranean Region, by country, in 2015. (Global Burden of Disease Study 2015, Eastern Mediterranean Countries, 2015).


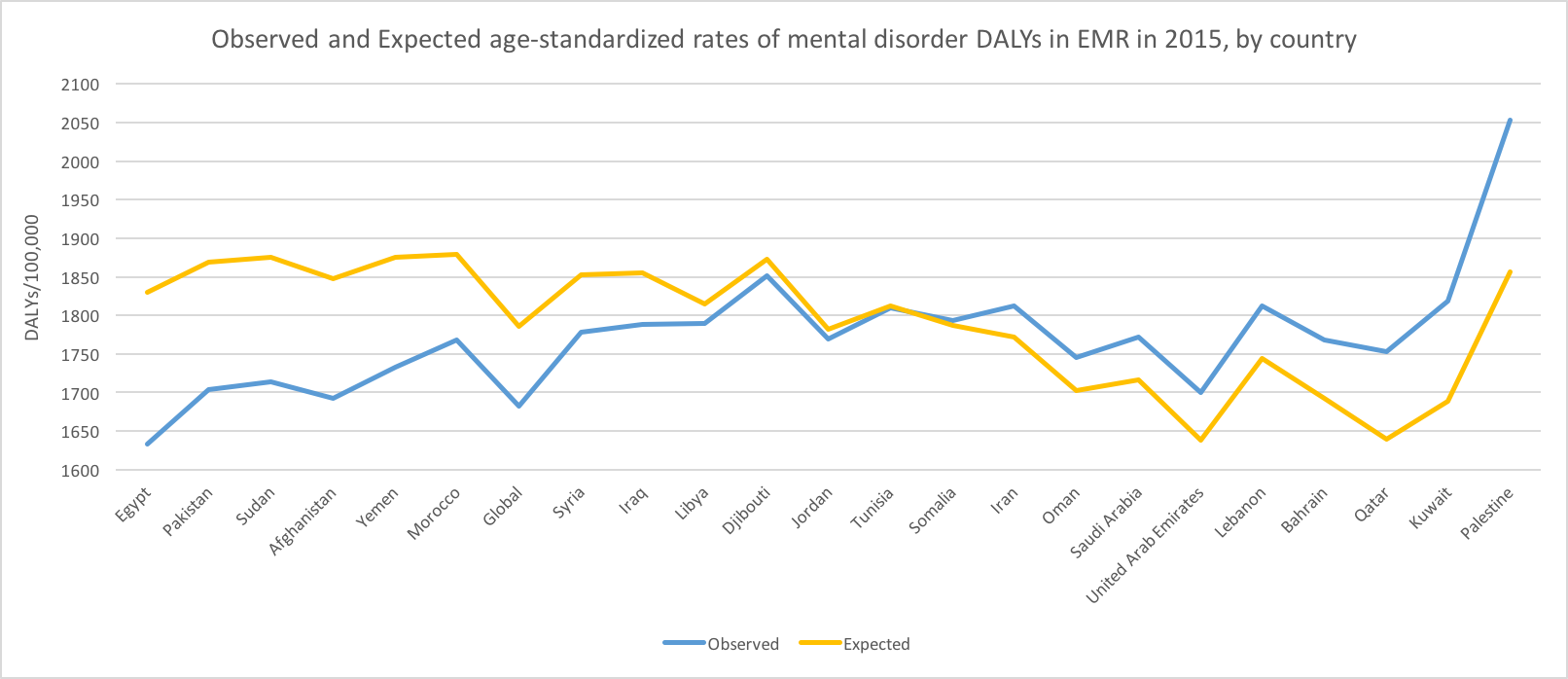


e-Table 1 – Age-standardized rates of disability-adjusted life-years(DALYs) per 100,000 population for mental disorders in the Eastern Mediterranean Region by age, sex, and disorder, in 2015. (Global Burden of Disease Study 2015, Eastern Mediterranean Region, 2015).


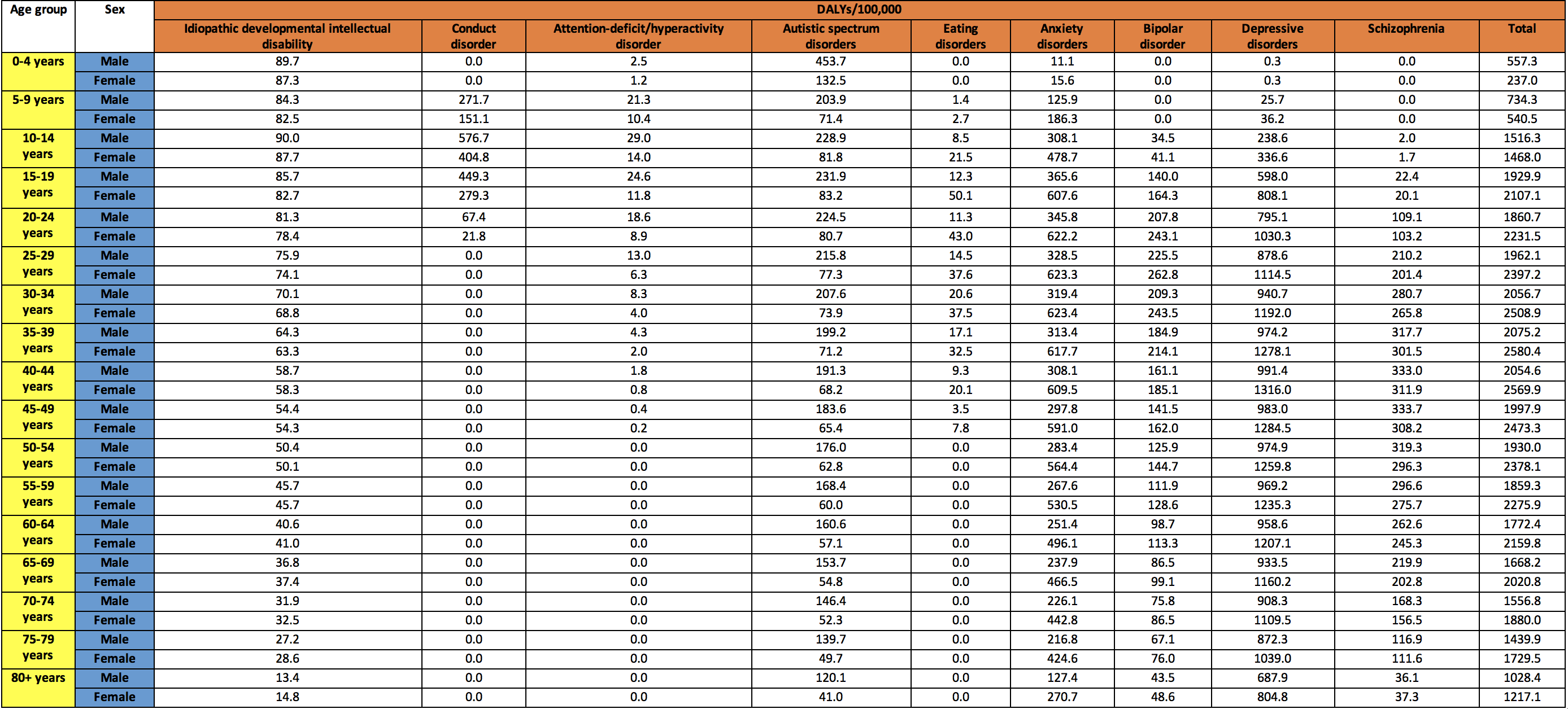


e-Table 2 – Age-standardized rates of disability-adjusted life-years(DALYs) per 100,000 population for mental disorders in the Eastern Mediterranean Region and globally, by year and sex, 1990-2015. EMR = Eastern Mediterranean Region. 95% uncertainty intervals included in parentheses. (Global Burden of Disease Study 2015, Global, Eastern Mediterranean Region, 1990-2015).
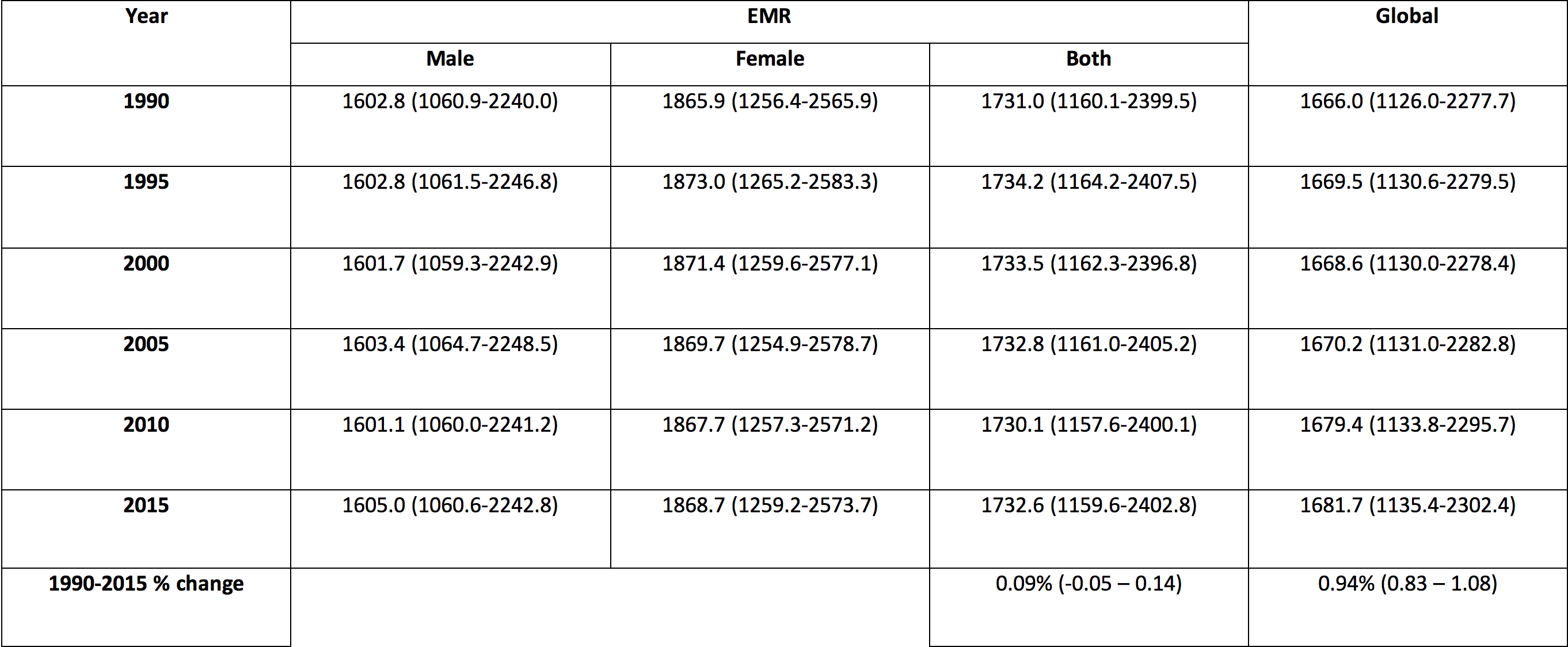


e-Table 3 – Age-standardized rates of disability-adjusted life-years(DALYs) per 100,000 population for mental disorders in the Eastern Mediterranean Region and globally, by income group and sex, 1990-2015. EMR = Eastern Mediterranean Region. (Global Burden of Disease Study 2015, Global, Eastern Mediterranean Region, 2015).


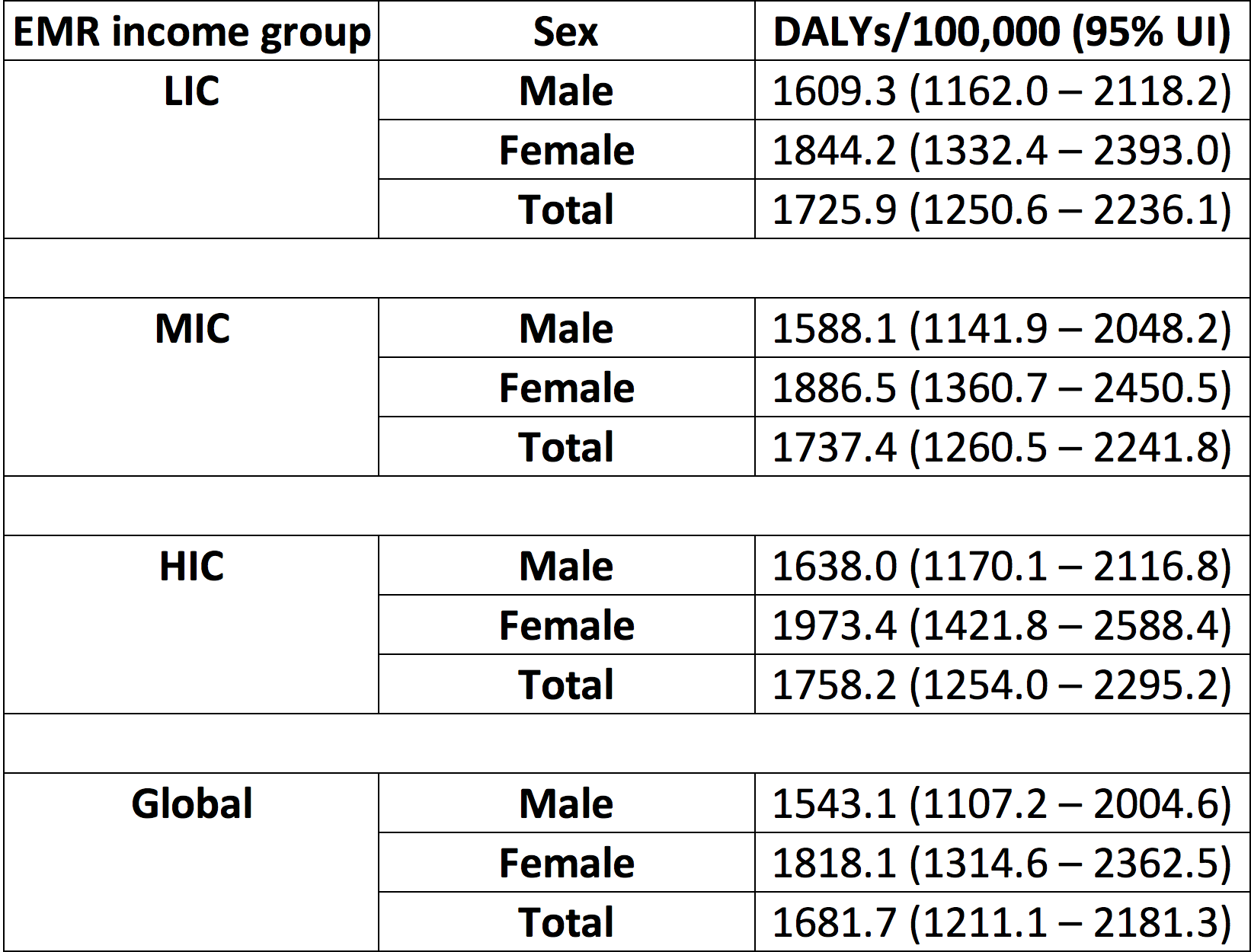

Supplement: Supplementary file 3 — Supplementary material 3 (DOCX 1189 kb) [file 38_2017_1006_MOESM3_ESM.docx]
