# Supplementary material for: The burden of mental disorders in the Eastern Mediterranean region, 1990–2015: findings from the global burden of disease 2015 study
Source: Int J Public Health. 2017 Aug 3;63(Suppl 1):25–37. doi: 10.1007/s00038-017-1006-1 (PMC5973970; doi:10.1007/s00038-017-1006-1)
Supplement: Supplementary file 1 — Supplementary material 1 (PDF 79 kb) [file 38_2017_1006_MOESM1_ESM.pdf]

Citation	Location	Cause	Data Collection Method	Year Start	Year End	Sex	Age Start	Age End	Representativeness	Urbanicity Type	Population Representativeness Covariates	Sample Size (people)	Standard Error
Al-Jawadi AA, Abdul-Rhman S. Prevalence of childhood and early adolescence mental disorders among children attending primary health care centers in Mosul, Iraq: a cross-sectional study. BMC Public Health. 2007; 7: 274	Iraq	ADHD	Unidentifiable	2003	2004	Females	5	9	Representative for subnational location only	Unknown	1339=0, 1338=0, 239=1, 1188=1, 1337=0		0.00627383
Eapen V, Jakka ME, Abou-Saleh MT. Children With Psychiatric Disorders: The Al Ain Community Psychiatric Survey. Can J Psychiatry. 2003; 48(6): 402-7	UAE	ADHD	Unidentifiable	2001	2001	Both sexes	6	18	Representative for subnational location only	Unknown	1338=0, 239=1, 1188=0, 1337=0, 1339=0	329	0.00602332
Eapen V, Al-Gazali L, Bin-Othman S, Abou-Saleh M. Mental Health Problems Among Schoolchildren in United Arab Emirates: Prevalence and Risk Factors. J Am Acad Child Adolesc Psychiatry. 1998; 37(8): 880-6	UAE	ADHD	Unidentifiable	1995	1996	Both sexes	6	15	Representative for subnational location only	Unknown	1338=0, 239=1, 1188=0, 1337=0, 1339=0	3278	0.0012191
Al-Jawadi AA, Abdul-Rhman S. Prevalence of childhood and early adolescence mental disorders among children attending primary health care centers in Mosul, Iraq: a cross-sectional study. BMC Public Health. 2007; 7: 274	Iraq	ADHD	Unidentifiable	2003	2004	Males	1	4	Representative for subnational location only	Unknown	1338=0, 239=1, 1188=1, 1337=0, 1339=0		0.00214438
Talaei A, Mokhber N, Abdollahian E, Bordbar MRF, Salari E. Attention deficit/hyperactivity disorder: a survey on prevalence rate among male subjects in elementary school (7 to 9 years old) in Iran. J Atten Disord. 2010; 13(4): 386-90	Iran	ADHD	Unidentifiable	2008	2008	Males	7	9	Representative for subnational location only	Urban	1339=0, 1338=0, 239=1, 1188=1, 1337=0	714	0.01353115
Al-Jawadi AA, Abdul-Rhman S. Prevalence of childhood and early adolescence mental disorders among children attending primary health care centers in Mosul, Iraq: a cross-sectional study. BMC Public Health. 2007; 7: 274	Iraq	ADHD	Unidentifiable	2003	2004	Males	5	9	Representative for subnational location only	Unknown	1339=0, 1338=0, 239=1, 1188=1, 1337=0		0.00520369
Al-Jawadi AA, Abdul-Rhman S. Prevalence of childhood and early adolescence mental disorders among children attending primary health care centers in Mosul, Iraq: a cross-sectional study. BMC Public Health. 2007; 7: 274	Iraq	ADHD	Unidentifiable	2003	2004	Males	10	15	Representative for subnational location only	Unknown	1188=1, 1337=0, 1339=0, 1338=0, 239=1		0.00917822
Amiri S, Fakhari A, Maheri M, Mohammadpoor Asl A. Attention deficit/hyperactivity disorder in primary school children of Tabriz, North-West Iran. Paediatr Perinat Epidemiol. 2010; 24(6): 597-601	Iran	ADHD	Unidentifiable	2007	2008	Males	7	15	Representative for subnational location only	Urban	239=1, 1188=1, 1337=0, 1339=0, 1338=0	877	0.011108
Amiri S, Fakhari A, Maheri M, Mohammadpoor Asl A. Attention deficit/hyperactivity disorder in primary school children of Tabriz, North-West Iran. Paediatr Perinat Epidemiol. 2010; 24(6): 597-601	Iran	ADHD	Unidentifiable	2007	2008	Females	7	15	Representative for subnational location only	Urban	1338=0, 239=1, 1188=1, 1337=0, 1339=0	781	0.00945057
Al-Jawadi AA, Abdul-Rhman S. Prevalence of childhood and early adolescence mental disorders among children attending primary health care centers in Mosul, Iraq: a cross-sectional study. BMC Public Health. 2007; 7: 274	Iraq	ADHD	Unidentifiable	2003	2004	Females	10	15	Representative for subnational location only	Unknown	1337=0, 1339=0, 1338=0, 239=1, 1188=1		0.01078233
Alyahri A, Goodman R. The prevalence of DSM-IV psychiatric disorders among 7-10 year old Yemeni schoolchildren. Soc Psychiatry Psychiatr Epidemiol. 2008; 43(3): 224-30	Yemen	ADHD	Unidentifiable	2002	2003	Males	7	10	Representative for subnational location only	Mixed/both	1337=0, 1339=0, 1338=0, 239=1, 1188=0	608	0.00603424
Alyahri A, Goodman R. The prevalence of DSM-IV psychiatric disorders among 7-10 year old Yemeni schoolchildren. Soc Psychiatry Psychiatr Epidemiol. 2008; 43(3): 224-30	Yemen	ADHD	Unidentifiable	2002	2003	Females	7	10	Representative for subnational location only	Mixed/both	1188=0, 1337=0, 1339=0, 1338=0, 239=1	602	0.00330365
Al-Jawadi AA, Abdul-Rhman S. Prevalence of childhood and early adolescence mental disorders among children attending primary health care centers in Mosul, Iraq: a cross-sectional study. BMC Public Health. 2007; 7: 274	Iraq	ADHD	Unidentifiable	2003	2004	Females	1	4	Representative for subnational location only	Unknown	1338=0, 239=1, 1188=1, 1337=0, 1339=0		0.00590351
World Health Organization (WHO). WHO World Mental Health Surveys: Global Perspectives on the Epidemiology of Mental Disorders. Cambridge, United Kingdom: Cambridge University Press, 2008	Iraq	Anxiety disorders	Unidentifiable	2006	2007	Females	65	69	Nationally representative only	Mixed/both	246=0, 1315=0, 1337=0, 1338=0, 1339=0, 1344=0	45	0.03671525
World Health Organization (WHO). WHO World Mental Health Surveys: Global Perspectives on the Epidemiology of Mental Disorders. Cambridge, United Kingdom: Cambridge University Press, 2008	Lebanon	Anxiety disorders	Unidentifiable	2002	2003	Males	35	39	Nationally representative only	Mixed/both	246=0, 1315=0, 1337=0, 1338=0, 1339=0, 1344=0	147	0.0125747
World Health Organization (WHO). WHO World Mental Health Surveys: Global Perspectives on the Epidemiology of Mental Disorders. Cambridge, United Kingdom: Cambridge University Press, 2008	Lebanon	Anxiety disorders	Unidentifiable	2002	2003	Males	40	44	Nationally representative only	Mixed/both	246=0, 1315=0, 1337=0, 1338=0, 1339=0, 1344=0	140	0.01723032
Ghubash R, Hamdi E, Bebbington P. The Dubai Community Psychiatric Survey: I. Prevalence and socio-demographic correlates. Soc Psychiatry Psychiatr Epidemiol. 1992; 27(2): 53-61	UAE	Anxiety disorders	Unidentifiable	1989	1990	Females	16	64	Representative for subnational location only	Mixed/both	246=1, 1315=0, 1337=0, 1338=0, 1339=0, 1344=0	300	0.01508876
World Health Organization (WHO). WHO World Mental Health Surveys: Global Perspectives on the Epidemiology of Mental Disorders. Cambridge, United Kingdom: Cambridge University Press, 2008	Lebanon	Anxiety disorders	Unidentifiable	2002	2003	Males	45	49	Nationally representative only	Mixed/both	246=0, 1315=0, 1337=0, 1338=0, 1339=0, 1344=0	133	0.01257303
World Health Organization (WHO). WHO World Mental Health Surveys: Global Perspectives on the Epidemiology of Mental Disorders. Cambridge, United Kingdom: Cambridge University Press, 2008	Lebanon	Anxiety disorders	Unidentifiable	2002	2003	Males	50	54	Nationally representative only	Mixed/both	246=0, 1315=0, 1337=0, 1338=0, 1339=0, 1344=0	96	0.02559049
World Health Organization (WHO). WHO World Mental Health Surveys: Global Perspectives on the Epidemiology of Mental Disorders. Cambridge, United Kingdom: Cambridge University Press, 2008	Lebanon	Anxiety disorders	Unidentifiable	2002	2003	Males	55	59	Nationally representative only	Mixed/both	246=0, 1315=0, 1337=0, 1338=0, 1339=0, 1344=0	69	0.03172283
World Health Organization (WHO). WHO World Mental Health Surveys: Global Perspectives on the Epidemiology of Mental Disorders. Cambridge, United Kingdom: Cambridge University Press, 2008	Lebanon	Anxiety disorders	Unidentifiable	2002	2003	Males	60	64	Nationally representative only	Mixed/both	246=0, 1315=0, 1337=0, 1338=0, 1339=0, 1344=0	77	0.01547359
World Health Organization (WHO). WHO World Mental Health Surveys: Global Perspectives on the Epidemiology of Mental Disorders. Cambridge, United Kingdom: Cambridge University Press, 2008	Lebanon	Anxiety disorders	Unidentifiable	2002	2003	Males	65	69	Nationally representative only	Mixed/both	246=0, 1315=0, 1337=0, 1338=0, 1339=0, 1344=0	70	0.0340987
World Health Organization (WHO). WHO World Mental Health Surveys: Global Perspectives on the Epidemiology of Mental Disorders. Cambridge, United Kingdom: Cambridge University Press, 2008	Lebanon	Anxiety disorders	Unidentifiable	2002	2003	Males	70	74	Nationally representative only	Mixed/both	246=0, 1315=0, 1337=0, 1338=0, 1339=0, 1344=0	70	0.01399974
World Health Organization (WHO). WHO World Mental Health Surveys: Global Perspectives on the Epidemiology of Mental Disorders. Cambridge, United Kingdom: Cambridge University Press, 2008	Lebanon	Anxiety disorders	Unidentifiable	2002	2003	Females	45	49	Nationally representative only	Mixed/both	246=0, 1315=0, 1337=0, 1338=0, 1339=0, 1344=0	110	0.02481964
World Health Organization (WHO). WHO World Mental Health Surveys: Global Perspectives on the Epidemiology of Mental Disorders. Cambridge, United Kingdom: Cambridge University Press, 2008	Lebanon	Anxiety disorders	Unidentifiable	2002	2003	Males	75	79	Nationally representative only	Mixed/both	246=0, 1315=0, 1337=0, 1338=0, 1339=0, 1344=0	41	0.023902
World Health Organization (WHO). WHO World Mental Health Surveys: Global Perspectives on the Epidemiology of Mental Disorders. Cambridge, United Kingdom: Cambridge University Press, 2008	Lebanon	Anxiety disorders	Unidentifiable	2002	2003	Females	30	34	Nationally representative only	Mixed/both	246=0, 1315=0, 1337=0, 1338=0, 1339=0, 1344=0	171	0.02163698
World Health Organization (WHO). WHO World Mental Health Surveys: Global Perspectives on the Epidemiology of Mental Disorders. Cambridge, United Kingdom: Cambridge University Press, 2008	Iraq	Anxiety disorders	Unidentifiable	2006	2007	Males	60	64	Nationally representative only	Mixed/both	246=0, 1315=0, 1337=0, 1338=0, 1339=0, 1344=0	52	0.0273958
World Health Organization (WHO). WHO World Mental Health Surveys: Global Perspectives on the Epidemiology of Mental Disorders. Cambridge, United Kingdom: Cambridge University Press, 2008	Lebanon	Anxiety disorders	Unidentifiable	2002	2003	Females	20	24	Nationally representative only	Mixed/both	246=0, 1315=0, 1337=0, 1338=0, 1339=0, 1344=0	168	0.02184298
World Health Organization (WHO). WHO World Mental Health Surveys: Global Perspectives on the Epidemiology of Mental Disorders. Cambridge, United Kingdom: Cambridge University Press, 2008	Iraq	Anxiety disorders	Unidentifiable	2006	2007	Males	40	44	Nationally representative only	Mixed/both	246=0, 1315=0, 1337=0, 1338=0, 1339=0, 1344=0	229	0.01348989
World Health Organization (WHO). WHO World Mental Health Surveys: Global Perspectives on the Epidemiology of Mental Disorders. Cambridge, United Kingdom: Cambridge University Press, 2008	Lebanon	Anxiety disorders	Unidentifiable	2002	2003	Females	25	29	Nationally representative only	Mixed/both	246=0, 1315=0, 1337=0, 1338=0, 1339=0, 1344=0	146	0.0269492
World Health Organization (WHO). WHO World Mental Health Surveys: Global Perspectives on the Epidemiology of Mental Disorders. Cambridge, United Kingdom: Cambridge University Press, 2008	Lebanon	Anxiety disorders	Unidentifiable	2002	2003	Females	40	44	Nationally representative only	Mixed/both	246=0, 1315=0, 1337=0, 1338=0, 1339=0, 1344=0	179	0.0212252
World Health Organization (WHO). WHO World Mental Health Surveys: Global Perspectives on the Epidemiology of Mental Disorders. Cambridge, United Kingdom: Cambridge University Press, 2008	Lebanon	Anxiety disorders	Unidentifiable	2002	2003	Females	50	54	Nationally representative only	Mixed/both	246=0, 1315=0, 1337=0, 1338=0, 1339=0, 1344=0	110	0.0285736
World Health Organization (WHO). WHO World Mental Health Surveys: Global Perspectives on the Epidemiology of Mental Disorders. Cambridge, United Kingdom: Cambridge University Press, 2008	Lebanon	Anxiety disorders	Unidentifiable	2002	2003	Females	55	59	Nationally representative only	Mixed/both	246=0, 1315=0, 1337=0, 1338=0, 1339=0, 1344=0	93	0.01474752
World Health Organization (WHO). WHO World Mental Health Surveys: Global Perspectives on the Epidemiology of Mental Disorders. Cambridge, United Kingdom: Cambridge University Press, 2008	Lebanon	Anxiety disorders	Unidentifiable	2002	2003	Females	60	64	Nationally representative only	Mixed/both	246=0, 1315=0, 1337=0, 1338=0, 1339=0, 1344=0	108	0.02408382
World Health Organization (WHO). WHO World Mental Health Surveys: Global Perspectives on the Epidemiology of Mental Disorders. Cambridge, United Kingdom: Cambridge University Press, 2008	Lebanon	Anxiety disorders	Unidentifiable	2002	2003	Females	65	69	Nationally representative only	Mixed/both	246=0, 1315=0, 1337=0, 1338=0, 1339=0, 1344=0	70	0.04243962
Ghanem M, Gadallah M, Meky FA, Mourad S, El-Kholy G. National Survey of Prevalence of Mental Disorders in Egypt: preliminary survey. East Mediterr Health J. 2009; 15(1): 65-75	Egypt	Anxiety disorders	Unidentifiable	2003	2003	Both sexes	18	64	Nationally representative only	Mixed/both	246=0, 1315=0, 1337=0, 1338=0, 1339=0, 1344=0	14640	0.00175923
World Health Organization (WHO). WHO World Mental Health Surveys: Global Perspectives on the Epidemiology of Mental Disorders. Cambridge, United Kingdom: Cambridge University Press, 2008	Lebanon	Anxiety disorders	Unidentifiable	2002	2003	Females	70	74	Nationally representative only	Mixed/both	246=0, 1315=0, 1337=0, 1338=0, 1339=0, 1344=0	60	0.03734458
World Health Organization (WHO). WHO World Mental Health Surveys: Global Perspectives on the Epidemiology of Mental Disorders. Cambridge, United Kingdom: Cambridge University Press, 2008	Iraq	Anxiety disorders	Unidentifiable	2006	2007	Females	25	29	Nationally representative only	Mixed/both	246=0, 1315=0, 1337=0, 1338=0, 1339=0, 1344=0	321	0.01752104
World Health Organization (WHO). WHO World Mental Health Surveys: Global Perspectives on the Epidemiology of Mental Disorders. Cambridge, United Kingdom: Cambridge University Press, 2008	Lebanon	Anxiety disorders	Unidentifiable	2002	2003	Females	75	79	Nationally representative only	Mixed/both	246=0, 1315=0, 1337=0, 1338=0, 1339=0, 1344=0	45	0.05640674
World Health Organization (WHO). WHO World Mental Health Surveys: Global Perspectives on the Epidemiology of Mental Disorders. Cambridge, United Kingdom: Cambridge University Press, 2008	Iraq	Anxiety disorders	Unidentifiable	2006	2007	Females	40	44	Nationally representative only	Mixed/both	246=0, 1315=0, 1337=0, 1338=0, 1339=0, 1344=0	219	0.02067185
World Health Organization (WHO). WHO World Mental Health Surveys: Global Perspectives on the Epidemiology of Mental Disorders. Cambridge, United Kingdom: Cambridge University Press, 2008	Iraq	Anxiety disorders	Unidentifiable	2006	2007	Males	18	18	Nationally representative only	Mixed/both	246=0, 1315=0, 1337=0, 1338=0, 1339=0, 1344=0	53	0.04615071
World Health Organization (WHO). WHO World Mental Health Surveys: Global Perspectives on the Epidemiology of Mental Disorders. Cambridge, United Kingdom: Cambridge University Press, 2008	Iraq	Anxiety disorders	Unidentifiable	2006	2007	Males	19	19	Nationally representative only	Mixed/both	246=0, 1315=0, 1337=0, 1338=0, 1339=0, 1344=0	68	0.02943438
World Health Organization (WHO). WHO World Mental Health Surveys: Global Perspectives on the Epidemiology of Mental Disorders. Cambridge, United Kingdom: Cambridge University Press, 2008	Iraq	Anxiety disorders	Unidentifiable	2006	2007	Males	20	24	Nationally representative only	Mixed/both	246=0, 1315=0, 1337=0, 1338=0, 1339=0, 1344=0	301	0.00940322
World Health Organization (WHO). WHO World Mental Health Surveys: Global Perspectives on the Epidemiology of Mental Disorders. Cambridge, United Kingdom: Cambridge University Press, 2008	Iraq	Anxiety disorders	Unidentifiable	2006	2007	Males	25	29	Nationally representative only	Mixed/both	246=0, 1315=0, 1337=0, 1338=0, 1339=0, 1344=0	284	0.01099897
World Health Organization (WHO). WHO World Mental Health Surveys: Global Perspectives on the Epidemiology of Mental Disorders. Cambridge, United Kingdom: Cambridge University Press, 2008	Iraq	Anxiety disorders	Unidentifiable	2006	2007	Males	30	34	Nationally representative only	Mixed/both	246=0, 1315=0, 1337=0, 1338=0, 1339=0, 1344=0	300	0.00752888
World Health Organization (WHO). WHO World Mental Health Surveys: Global Perspectives on the Epidemiology of Mental Disorders. Cambridge, United Kingdom: Cambridge University Press, 2008	Iraq	Anxiety disorders	Unidentifiable	2006	2007	Males	35	39	Nationally representative only	Mixed/both	246=0, 1315=0, 1337=0, 1338=0, 1339=0, 1344=0	310	0.00957516
World Health Organization (WHO). WHO World Mental Health Surveys: Global Perspectives on the Epidemiology of Mental Disorders. Cambridge, United Kingdom: Cambridge University Press, 2008	Lebanon	Anxiety disorders	Unidentifiable	2002	2003	Females	35	39	Nationally representative only	Mixed/both	246=0, 1315=0, 1337=0, 1338=0, 1339=0, 1344=0	222	0.02170201
World Health Organization (WHO). WHO World Mental Health Surveys: Global Perspectives on the Epidemiology of Mental Disorders. Cambridge, United Kingdom: Cambridge University Press, 2008	Iraq	Anxiety disorders	Unidentifiable	2006	2007	Males	45	49	Nationally representative only	Mixed/both	246=0, 1315=0, 1337=0, 1338=0, 1339=0, 1344=0	137	0.01961415
World Health Organization (WHO). WHO World Mental Health Surveys: Global Perspectives on the Epidemiology of Mental Disorders. Cambridge, United Kingdom: Cambridge University Press, 2008	Iraq	Anxiety disorders	Unidentifiable	2006	2007	Males	50	54	Nationally representative only	Mixed/both	246=0, 1315=0, 1337=0, 1338=0, 1339=0, 1344=0	129	0.00941246
World Health Organization (WHO). WHO World Mental Health Surveys: Global Perspectives on the Epidemiology of Mental Disorders. Cambridge, United Kingdom: Cambridge University Press, 2008	Iraq	Anxiety disorders	Unidentifiable	2006	2007	Males	55	59	Nationally representative only	Mixed/both	246=0, 1315=0, 1337=0, 1338=0, 1339=0, 1344=0	103	0.01330849
World Health Organization (WHO). WHO World Mental Health Surveys: Global Perspectives on the Epidemiology of Mental Disorders. Cambridge, United Kingdom: Cambridge University Press, 2008	Lebanon	Anxiety disorders	Unidentifiable	2002	2003	Males	20	24	Nationally representative only	Mixed/both	246=0, 1315=0, 1337=0, 1338=0, 1339=0, 1344=0	125	0.01715411
World Health Organization (WHO). WHO World Mental Health Surveys: Global Perspectives on the Epidemiology of Mental Disorders. Cambridge, United Kingdom: Cambridge University Press, 2008	Iraq	Anxiety disorders	Unidentifiable	2006	2007	Males	65	69	Nationally representative only	Mixed/both	246=0, 1315=0, 1337=0, 1338=0, 1339=0, 1344=0	55	0.02308765
World Health Organization (WHO). WHO World Mental Health Surveys: Global Perspectives on the Epidemiology of Mental Disorders. Cambridge, United Kingdom: Cambridge University Press, 2008	Iraq	Anxiety disorders	Unidentifiable	2006	2007	Females	18	18	Nationally representative only	Mixed/both	246=0, 1315=0, 1337=0, 1338=0, 1339=0, 1344=0	69	0.04678386
World Health Organization (WHO). WHO World Mental Health Surveys: Global Perspectives on the Epidemiology of Mental Disorders. Cambridge, United Kingdom: Cambridge University Press, 2008	Iraq	Anxiety disorders	Unidentifiable	2006	2007	Females	19	19	Nationally representative only	Mixed/both	246=0, 1315=0, 1337=0, 1338=0, 1339=0, 1344=0	55	0.04625859
World Health Organization (WHO). WHO World Mental Health Surveys: Global Perspectives on the Epidemiology of Mental Disorders. Cambridge, United Kingdom: Cambridge University Press, 2008	Iraq	Anxiety disorders	Unidentifiable	2006	2007	Females	20	24	Nationally representative only	Mixed/both	246=0, 1315=0, 1337=0, 1338=0, 1339=0, 1344=0	319	0.01757755
World Health Organization (WHO). WHO World Mental Health Surveys: Global Perspectives on the Epidemiology of Mental Disorders. Cambridge, United Kingdom: Cambridge University Press, 2008	Lebanon	Anxiety disorders	Unidentifiable	2002	2003	Males	25	29	Nationally representative only	Mixed/both	246=0, 1315=0, 1337=0, 1338=0, 1339=0, 1344=0	114	0.01986363
World Health Organization (WHO). WHO World Mental Health Surveys: Global Perspectives on the Epidemiology of Mental Disorders. Cambridge, United Kingdom: Cambridge University Press, 2008	Iraq	Anxiety disorders	Unidentifiable	2006	2007	Females	30	34	Nationally representative only	Mixed/both	246=0, 1315=0, 1337=0, 1338=0, 1339=0, 1344=0	378	0.01040714
World Health Organization (WHO). WHO World Mental Health Surveys: Global Perspectives on the Epidemiology of Mental Disorders. Cambridge, United Kingdom: Cambridge University Press, 2008	Iraq	Anxiety disorders	Unidentifiable	2006	2007	Females	35	39	Nationally representative only	Mixed/both	246=0, 1315=0, 1337=0, 1338=0, 1339=0, 1344=0	321	0.01594342
World Health Organization (WHO). WHO World Mental Health Surveys: Global Perspectives on the Epidemiology of Mental Disorders. Cambridge, United Kingdom: Cambridge University Press, 2008	Lebanon	Anxiety disorders	Unidentifiable	2002	2003	Males	30	34	Nationally representative only	Mixed/both	246=0, 1315=0, 1337=0, 1338=0, 1339=0, 1344=0	134	0.01687153
World Health Organization (WHO). WHO World Mental Health Surveys: Global Perspectives on the Epidemiology of Mental Disorders. Cambridge, United Kingdom: Cambridge University Press, 2008	Iraq	Anxiety disorders	Unidentifiable	2006	2007	Females	45	49	Nationally representative only	Mixed/both	246=0, 1315=0, 1337=0, 1338=0, 1339=0, 1344=0	116	0.01772345
World Health Organization (WHO). WHO World Mental Health Surveys: Global Perspectives on the Epidemiology of Mental Disorders. Cambridge, United Kingdom: Cambridge University Press, 2008	Iraq	Anxiety disorders	Unidentifiable	2006	2007	Females	50	54	Nationally representative only	Mixed/both	246=0, 1315=0, 1337=0, 1338=0, 1339=0, 1344=0	138	0.02229629
World Health Organization (WHO). WHO World Mental Health Surveys: Global Perspectives on the Epidemiology of Mental Disorders. Cambridge, United Kingdom: Cambridge University Press, 2008	Iraq	Anxiety disorders	Unidentifiable	2006	2007	Females	55	59	Nationally representative only	Mixed/both	246=0, 1315=0, 1337=0, 1338=0, 1339=0, 1344=0	101	0.02356948
World Health Organization (WHO). WHO World Mental Health Surveys: Global Perspectives on the Epidemiology of Mental Disorders. Cambridge, United Kingdom: Cambridge University Press, 2008	Iraq	Anxiety disorders	Unidentifiable	2006	2007	Females	60	64	Nationally representative only	Mixed/both	246=0, 1315=0, 1337=0, 1338=0, 1339=0, 1344=0	66	0.02515617
Al-Jawadi AA, Abdul-Rhman S. Prevalence of childhood and early adolescence mental disorders among children attending primary health care centers in Mosul, Iraq: a cross-sectional study. BMC Public Health. 2007; 7: 274	Iraq	Autism	Unidentifiable	2003	2004	Males	5	9	Representative for subnational location only	Urban	1186=0, 1337=0, 1338=0, 1339=0		0.00192578
Al-Jawadi AA, Abdul-Rhman S. Prevalence of childhood and early adolescence mental disorders among children attending primary health care centers in Mosul, Iraq: a cross-sectional study. BMC Public Health. 2007; 7: 274	Iraq	Autism	Unidentifiable	2003	2004	Males	10	15	Representative for subnational location only	Urban	1186=0, 1337=0, 1338=0, 1339=0		0.00264352
Al-Jawadi AA, Abdul-Rhman S. Prevalence of childhood and early adolescence mental disorders among children attending primary health care centers in Mosul, Iraq: a cross-sectional study. BMC Public Health. 2007; 7: 274	Iraq	Autism	Unidentifiable	2003	2004	Females	1	4	Representative for subnational location only	Urban	1186=0, 1337=0, 1338=0, 1339=0		0.00313525
Al-Jawadi AA, Abdul-Rhman S. Prevalence of childhood and early adolescence mental disorders among children attending primary health care centers in Mosul, Iraq: a cross-sectional study. BMC Public Health. 2007; 7: 274	Iraq	Autism	Unidentifiable	2003	2004	Females	5	9	Representative for subnational location only	Urban	1186=0, 1337=0, 1338=0, 1339=0		0.00221751
Al-Jawadi AA, Abdul-Rhman S. Prevalence of childhood and early adolescence mental disorders among children attending primary health care centers in Mosul, Iraq: a cross-sectional study. BMC Public Health. 2007; 7: 274	Iraq	Autism	Unidentifiable	2003	2004	Females	10	15	Representative for subnational location only	Urban	1186=0, 1337=0, 1338=0, 1339=0		0.00313525
Samadi SA, Mahmoodizadeh A, McConkey R. A national study of the prevalence of autism among five-year-old children in Iran. Autism. 2012; 16(1): 5-14	Iran	Autism	Unidentifiable	2006	2009	Females	5	6	Nationally representative only	Urban	1186=1, 1337=0, 1338=0, 1339=0	643442.625	2.1429E-05
Samadi SA, Mahmoodizadeh A, McConkey R. A national study of the prevalence of autism among five-year-old children in Iran. Autism. 2012; 16(1): 5-14	Iran	Autism	Unidentifiable	2006	2009	Males	5	6	Nationally representative only	Urban	1186=1, 1337=0, 1338=0, 1339=0	678499	3.813E-05
Al-Jawadi AA, Abdul-Rhman S. Prevalence of childhood and early adolescence mental disorders among children attending primary health care centers in Mosul, Iraq: a cross-sectional study. BMC Public Health. 2007; 7: 274	Iraq	Autism	Unidentifiable	2003	2004	Males	1	4	Representative for subnational location only	Urban	1186=0, 1337=0, 1338=0, 1339=0		0.00264352
Alhasnawi S, Sadik S, Rasheed M, Baban A, Al-Alak MM, Othman AY, Othman Y, Ismet N, Shawani O, Murthy S, Aljadiry M, Chatterji S, Al-Gasseer N, Streel E, Naidoo N, Mahomoud Ali M, Gruber MJ, Petukhova M, Sampson NA, Kessler RC, Iraq Mental Health Survey Study Group. The prevalence and correlates of DSM-IV disorders in the Iraq Mental Health Survey (IMHS). World Psychiatry. 2009; 8(2): 97-109	Iraq	Bipolar disorder	Unidentifiable	2006	2007	Both sexes	18	99	Nationally representative only	Mixed/both	1337=0, 1339=0, 214=0, 1315=0, 1338=0	4332	0.00071549
Karam EG, Mneimneh ZN, Karam AN, Fayyad JA, Nasser SC, Chatterji S, Kessler RC. Prevalence and treatment of mental disorders in Lebanon: a national epidemiological survey. Lancet. 2006; 367(9515): 1000-6	Lebanon	Bipolar disorder	Unidentifiable	2002	2003	Both sexes	18	99	Nationally representative only	Mixed/both	1338=0, 1337=0, 1339=0, 214=0, 1315=0	2857	0.00229982
Al-Jawadi AA, Abdul-Rhman S. Prevalence of childhood and early adolescence mental disorders among children attending primary health care centers in Mosul, Iraq: a cross-sectional study. BMC Public Health. 2007; 7: 274	Iraq	Conduct disorder	Unidentifiable	2003	2004	Females	10	15	Representative for subnational location only	Unknown	1338=0, 239=1, 1188=1, 1337=0, 1339=0	396.0451445	0.00690609
Alyahri A, Goodman R. The prevalence of DSM-IV psychiatric disorders among 7-10 year old Yemeni schoolchildren. Soc Psychiatry Psychiatr Epidemiol. 2008; 43(3): 224-30	Yemen	Conduct disorder	Unidentifiable	2002	2003	Both sexes	7	10	Representative for subnational location only	Mixed/both	239=1, 1188=0, 1337=0, 1339=0, 1338=0	1210	0.00663277
Eapen V, Al-Gazali L, Bin-Othman S, Abou-Saleh M. Mental Health Problems Among Schoolchildren in United Arab Emirates: Prevalence and Risk Factors. J Am Acad Child Adolesc Psychiatry. 1998; 37(8): 880-6	UAE	Conduct disorder	Unidentifiable	1995	1996	Both sexes	6	15	Representative for subnational location only	Unknown	239=1, 1188=1, 1337=0, 1339=0, 1338=0	3278	0.00274323
Al-Jawadi AA, Abdul-Rhman S. Prevalence of childhood and early adolescence mental disorders among children attending primary health care centers in Mosul, Iraq: a cross-sectional study. BMC Public Health. 2007; 7: 274	Iraq	Conduct disorder	Unidentifiable	2003	2004	Males	1	4	Representative for subnational location only	Unknown	1188=1, 1337=0, 1339=0, 1338=0, 239=1	457.1750568	0.00561921
Al-Jawadi AA, Abdul-Rhman S. Prevalence of childhood and early adolescence mental disorders among children attending primary health care centers in Mosul, Iraq: a cross-sectional study. BMC Public Health. 2007; 7: 274	Iraq	Conduct disorder	Unidentifiable	2003	2004	Males	5	9	Representative for subnational location only	Unknown	1337=0, 1339=0, 239=1, 1338=0, 1188=1	753.8700877	0.00688242
Al-Jawadi AA, Abdul-Rhman S. Prevalence of childhood and early adolescence mental disorders among children attending primary health care centers in Mosul, Iraq: a cross-sectional study. BMC Public Health. 2007; 7: 274	Iraq	Conduct disorder	Unidentifiable	2003	2004	Males	10	15	Representative for subnational location only	Unknown	239=1, 1338=0, 1188=1, 1337=0, 1339=0	486.9548555	0.01097418
Al-Jawadi AA, Abdul-Rhman S. Prevalence of childhood and early adolescence mental disorders among children attending primary health care centers in Mosul, Iraq: a cross-sectional study. BMC Public Health. 2007; 7: 274	Iraq	Conduct disorder	Unidentifiable	2003	2004	Females	1	4	Representative for subnational location only	Unknown	1337=0, 1339=0, 1338=0, 239=1, 1188=1	371.8249432	0.00403
Al-Jawadi AA, Abdul-Rhman S. Prevalence of childhood and early adolescence mental disorders among children attending primary health care centers in Mosul, Iraq: a cross-sectional study. BMC Public Health. 2007; 7: 274	Iraq	Conduct disorder	Unidentifiable	2003	2004	Females	5	9	Representative for subnational location only	Unknown	1338=0, 239=1, 1188=1, 1337=0, 1339=0	613.1299123	0.00430869
Madianos MG, Sarhan AL, Koukia E. Major depression across West Bank: a cross-sectional general population study. Int J Soc Psychiatry. 2012; 58(3): 315-22	Palestine	Major depressive disorder	Unidentifiable	2007	2007	Males	20	70	Representative for subnational location only	Mixed/both	214=0, 246=1, 1315=0, 1337=0, 1338=0, 1339=0, 1344=0	455	0.01499756
Madianos MG, Sarhan AL, Koukia E. Major depression across West Bank: a cross-sectional general population study. Int J Soc Psychiatry. 2012; 58(3): 315-22	Palestine	Major depressive disorder	Unidentifiable	2007	2007	Females	20	70	Representative for subnational location only	Mixed/both	214=0, 246=1, 1315=0, 1337=0, 1338=0, 1339=0, 1344=0	461	0.01394708
Ghanem M, Gadallah M, Meky FA, Mourad S, El-Kholy G. National Survey of Prevalence of Mental Disorders in Egypt: preliminary survey. East Mediterr Health J. 2009; 15(1): 65-75	Egypt	Dysthymia	Unidentifiable	2003	2003	Both sexes	18	65	Nationally representative only	Mixed/both	246=0, 1315=0, 214=0, 1338=0, 1344=0, 1337=0, 1339=0	14640	0.00083713
Al-Jawadi AA, Abdul-Rhman S. Prevalence of childhood and early adolescence mental disorders among children attending primary health care centers in Mosul, Iraq: a cross-sectional study. BMC Public Health. 2007; 7: 274	Iraq	Major depressive disorder	Unidentifiable	2003	2004	Males	1	4	Representative for subnational location only	Urban	214=1, 246=1, 1315=0, 1337=0, 1338=0, 1339=0, 1344=0		0.00621142
Al-Jawadi AA, Abdul-Rhman S. Prevalence of childhood and early adolescence mental disorders among children attending primary health care centers in Mosul, Iraq: a cross-sectional study. BMC Public Health. 2007; 7: 274	Iraq	Major depressive disorder	Unidentifiable	2003	2004	Males	5	9	Representative for subnational location only	Urban	214=1, 246=1, 1315=0, 1337=0, 1338=0, 1339=0, 1344=0		0.00542709
Al-Jawadi AA, Abdul-Rhman S. Prevalence of childhood and early adolescence mental disorders among children attending primary health care centers in Mosul, Iraq: a cross-sectional study. BMC Public Health. 2007; 7: 274	Iraq	Major depressive disorder	Unidentifiable	2003	2004	Males	10	15	Representative for subnational location only	Urban	214=1, 246=1, 1315=0, 1337=0, 1338=0, 1339=0, 1344=0		0.01146704
Al-Jawadi AA, Abdul-Rhman S. Prevalence of childhood and early adolescence mental disorders among children attending primary health care centers in Mosul, Iraq: a cross-sectional study. BMC Public Health. 2007; 7: 274	Iraq	Major depressive disorder	Unidentifiable	2003	2004	Females	1	4	Representative for subnational location only	Urban	214=1, 246=1, 1315=0, 1337=0, 1338=0, 1339=0, 1344=0		0.00812872
Al-Jawadi AA, Abdul-Rhman S. Prevalence of childhood and early adolescence mental disorders among children attending primary health care centers in Mosul, Iraq: a cross-sectional study. BMC Public Health. 2007; 7: 274	Iraq	Major depressive disorder	Unidentifiable	2003	2004	Females	5	9	Representative for subnational location only	Urban	214=1, 246=1, 1315=0, 1337=0, 1338=0, 1339=0, 1344=0		0.00734397
Al-Jawadi AA, Abdul-Rhman S. Prevalence of childhood and early adolescence mental disorders among children attending primary health care centers in Mosul, Iraq: a cross-sectional study. BMC Public Health. 2007; 7: 274	Iraq	Major depressive disorder	Unidentifiable	2003	2004	Females	10	15	Representative for subnational location only	Urban	214=1, 246=1, 1315=0, 1337=0, 1338=0, 1339=0, 1344=0		0.01567155
Ghanem M, Gadallah M, Meky FA, Mourad S, El-Kholy G. National Survey of Prevalence of Mental Disorders in Egypt: preliminary survey. East Mediterr Health J. 2009; 15(1): 65-75	Egypt	Major depressive disorder	Unidentifiable	2003	2003	Both sexes	18	65	Nationally representative only	Mixed/both	214=0, 246=0, 1315=0, 1337=0, 1338=0, 1339=0, 1344=0	14640	0.00134125
Farhood L, Zurayk H, Chaya M, Saadeh F, Meshefedjian G, Sidani T. The impact of war on the physical and mental health of the family: the Lebanese experience. Soc Sci Med. 1993; 36(12): 1555-67	Lebanon	Major depressive disorder	Unidentifiable	1987	1987	Both sexes	12	80	Representative for subnational location only	Urban	214=1, 246=1, 1315=0, 1337=0, 1338=0, 1339=0, 1344=0	1159	0.00881345
Farhood L, Zurayk H, Chaya M, Saadeh F, Meshefedjian G, Sidani T. The impact of war on the physical and mental health of the family: the Lebanese experience. Soc Sci Med. 1993; 36(12): 1555-67	Lebanon	Major depressive disorder	Unidentifiable	1987	1987	Both sexes	40	59	Representative for subnational location only	Urban	214=1, 246=1, 1315=0, 1337=0, 1338=0, 1339=0, 1344=0		0.0107001
Farhood LF, Dimassi H. Prevalence and predictors for post-traumatic stress disorder, depression and general health in a population from six villages in South Lebanon. Soc Psychiatry Psychiatr Epidemiol. 2012; 47(4): 639-49	Lebanon	Major depressive disorder	Unidentifiable	2010	2010	Both sexes	20	99	Representative for subnational location only	Urban	214=1, 246=1, 1315=0, 1337=0, 1338=0, 1339=0, 1344=0	632	0.01393012
Shaaban KMA, Baashar TA. A community study of depression in adolescent girls: prevalence and its relation to age. Med Princ Pract. 2003; 12(4): 256-9	Sudan	Major depressive disorder	Unidentifiable	2005	2005	Females	12	19	Representative for subnational location only	Urban	214=1, 246=1, 1315=0, 1337=0, 1338=0, 1339=0, 1344=0	1107	0.00609349
Kessler RC, Birnbaum HG, Shahly V, Bromet E, Hwang I, McLaughlin KA, Sampson N, Andrade LH, de Girolamo G, Demyttenaere K, Haro JM, Karam AN, Kostyuchenko S, Kovess V, Lara C, Levinson D, Matschinger H, Nakane Y, Browne MO, Ormel J, Posada-Villa J, Sagar R, Stein DJ. Age differences in the prevalence and co-morbidity of DSM-IV major depressive episodes: results from the WHO World Mental Health Survey Initiative. Depress Anxiety. 2010; 27(4): 351Â–64	Lebanon	Major depressive disorder	Survey - cross-sectional	2002	2003	Both sexes	18	34	Nationally representative only	Mixed/both	214=0, 246=0, 1315=0, 1337=0, 1338=0, 1339=0, 1344=0		0.011
Kessler RC, Birnbaum HG, Shahly V, Bromet E, Hwang I, McLaughlin KA, Sampson N, Andrade LH, de Girolamo G, Demyttenaere K, Haro JM, Karam AN, Kostyuchenko S, Kovess V, Lara C, Levinson D, Matschinger H, Nakane Y, Browne MO, Ormel J, Posada-Villa J, Sagar R, Stein DJ. Age differences in the prevalence and co-morbidity of DSM-IV major depressive episodes: results from the WHO World Mental Health Survey Initiative. Depress Anxiety. 2010; 27(4): 351Â–64	Lebanon	Major depressive disorder	Survey - cross-sectional	2002	2003	Both sexes	35	49	Nationally representative only	Mixed/both	214=0, 246=0, 1315=0, 1337=0, 1338=0, 1339=0, 1344=0		0.011
Karam EG, Mneimneh ZN, Karam AN, Fayyad JA, Nasser SC, Chatterji S, Kessler RC. Prevalence and treatment of mental disorders in Lebanon: a national epidemiological survey. Lancet. 2006; 367(9515): 1000-6	Lebanon	Dysthymia	Unidentifiable	2002	2003	Both sexes	18	99	Nationally representative only	Mixed/both	246=0, 214=0, 1315=0, 1338=0, 1344=0, 1337=0, 1339=0	2857	0.00170159
Kessler RC, Birnbaum HG, Shahly V, Bromet E, Hwang I, McLaughlin KA, Sampson N, Andrade LH, de Girolamo G, Demyttenaere K, Haro JM, Karam AN, Kostyuchenko S, Kovess V, Lara C, Levinson D, Matschinger H, Nakane Y, Browne MO, Ormel J, Posada-Villa J, Sagar R, Stein DJ. Age differences in the prevalence and co-morbidity of DSM-IV major depressive episodes: results from the WHO World Mental Health Survey Initiative. Depress Anxiety. 2010; 27(4): 351Â–64	Lebanon	Major depressive disorder	Survey - cross-sectional	2002	2003	Both sexes	50	64	Nationally representative only	Mixed/both	214=0, 246=0, 1315=0, 1337=0, 1338=0, 1339=0, 1344=0		0.01
Alhasnawi S, Sadik S, Rasheed M, Baban A, Al-Alak MM, Othman AY, Othman Y, Ismet N, Shawani O, Murthy S, Aljadiry M, Chatterji S, Al-Gasseer N, Streel E, Naidoo N, Mahomoud Ali M, Gruber MJ, Petukhova M, Sampson NA, Kessler RC, Iraq Mental Health Survey Study Group. The prevalence and correlates of DSM-IV disorders in the Iraq Mental Health Survey (IMHS). World Psychiatry. 2009; 8(2): 97-109	Iraq	Major depressive disorder	Unidentifiable	2006	2007	Both sexes	18	99	Nationally representative only	Mixed/both	214=0, 246=0, 1315=0, 1337=0, 1338=0, 1339=0, 1344=0	4332	0.00295006
Kessler RC, Birnbaum HG, Shahly V, Bromet E, Hwang I, McLaughlin KA, Sampson N, Andrade LH, de Girolamo G, Demyttenaere K, Haro JM, Karam AN, Kostyuchenko S, Kovess V, Lara C, Levinson D, Matschinger H, Nakane Y, Browne MO, Ormel J, Posada-Villa J, Sagar R, Stein DJ. Age differences in the prevalence and co-morbidity of DSM-IV major depressive episodes: results from the WHO World Mental Health Survey Initiative. Depress Anxiety. 2010; 27(4): 351Â–64	Lebanon	Major depressive disorder	Survey - cross-sectional	2002	2003	Both sexes	65	99	Nationally representative only	Mixed/both	214=0, 246=0, 1315=0, 1337=0, 1338=0, 1339=0, 1344=0		0.011
Nisar N, Billoo N, Gadit AA. Prevalence of depression and the associated risks factors among adult women in a fishing community. J Pak Med Assoc. 2004; 54(10): 519-25	Pakistan	Major depressive disorder	Unidentifiable	2000	2000	Females	18	99	Representative for subnational location only	Rural	214=1, 246=1, 1315=0, 1337=0, 1338=0, 1339=0, 1344=0	1200	0.00764718
Mumford DB, Nazir M, Jilani FU, Baig IY. Stress and psychiatric disorder in the Hindu Kush: a community survey of mountain villages in Chitral, Pakistan. Br J Psychiatry. 1996; 168(3): 299-307	Pakistan	Major depressive disorder	Unidentifiable	1994	1994	Males	18	80	Representative for subnational location only	Rural	214=1, 246=1, 1315=0, 1337=0, 1338=0, 1339=0, 1344=0	248	0.00563989
Mumford DB, Nazir M, Jilani FU, Baig IY. Stress and psychiatric disorder in the Hindu Kush: a community survey of mountain villages in Chitral, Pakistan. Br J Psychiatry. 1996; 168(3): 299-307	Pakistan	Major depressive disorder	Unidentifiable	1994	1994	Females	18	80	Representative for subnational location only	Rural	214=1, 246=1, 1315=0, 1337=0, 1338=0, 1339=0, 1344=0	326	0.01124641
Mousa TY, Al-Domi HA, Mashal RH, Jibril MA-K. Eating disturbances among adolescent schoolgirls in Jordan. Appetite. 2010; 54(1): 196-201	Jordan	Anorexia nervosa	Unidentifiable	2008	2008	Females	10	16	Representative for subnational location only	Mixed/both	1188=1, 1338=0, 217=0, 239=1, 1337=0, 208=0, 1339=0	326	0.00300608
Eapen V, Mabrouk AA, Bin-Othman S. Disordered eating attitudes and symptomatology among adolescent girls in the United Arab Emirates. Eat Behav. 2006; 7(1): 53-60	UAE	Anorexia nervosa	Unidentifiable	2003	2004	Females	13	18	Representative for subnational location only	Mixed/both	1339=0, 1188=0, 1338=0, 217=0, 239=1, 1337=0, 208=0	495	0.00282709
Eapen V, Mabrouk AA, Bin-Othman S. Disordered eating attitudes and symptomatology among adolescent girls in the United Arab Emirates. Eat Behav. 2006; 7(1): 53-60	UAE	Bulimia nervosa	Unidentifiable	2003	2004	Females	13	18	Representative for subnational location only	Mixed/both	208=0, 1339=0, 1188=0, 1338=0, 217=0, 239=1, 1337=0	495	0.00282709
Ahmadvand A, Sepehrmanesh Z, Ghoreishi FS, Afshinmajd S. Prevalence of psychiatric disorders in the general population of Kashan, Iran. Arch Iran Med. 2012; 15(4): 205	Iran	Schizophrenia	Survey - cross-sectional	2008	2009	Both sexes	18	99	Representative for subnational location only	Urban	1188=1	1800	0.00189993
